# Estimating per-infection cost and burden for dengue and Zika as a function of antibody-dependent enhancement

**DOI:** 10.1371/journal.pntd.0012876

**Published:** 2025-02-27

**Authors:** Christopher M. Kribs

**Affiliations:** Departments of Mathematics and Teacher & Administrator Preparation, University of Texas at Arlington, Arlington, Texas, United States of America; Imperial College London, UNITED KINGDOM OF GREAT BRITAIN AND NORTHERN IRELAND

## Abstract

The complex immune interactions produced by the tetravalent dengue vaccine Dengvaxia have foregrounded the important role of antibody-dependent enhancement (ADE) in dengue infection. Some evidence exists that ADE may extend beyond the four dengue serotypes to Zika, a closely related flavivirus transmitted by the same mosquito species as dengue, and may also account for the increased severity of some cases. Estimates of the public health impact of dengue vaccination may then need to include its effects on the transmission of Zika in addition to dengue. This study gathers primary references to build estimates of per-case economic cost and disease burden for dengue and Zika infection with and without ADE in the ten countries where clinical trials were held for Dengvaxia, under the hypothesis that severe outcomes are associated with ADE of disease. From these estimates, per-infection weighted averages are developed (without assumptions on transmission dynamics or case totals) which will facilitate population-level estimates of the potential impact of dengue vaccination on a dual outbreak using mathematical modeling. Results estimate that ADE amplifies the per-case toll of dengue by a factor of 2–16 but increases that of a Zika case by more than two orders of magnitude due to the greater risk of severe consequences. As expected, dengue vaccination affects per-infection dengue toll much more when high prior dengue seropositivity involves a different serotype than the one(s) circulating, but that same high dengue seropositivity makes vaccination exacerbate Zika toll less.

## Author summary

Dengue and Zika are closely related viruses carried by mosquitos which affect over 100 million people worldwide each year. Recovery from infection by one type produces immunity against that type, but makes later infections with other types of those viruses more severe, due to complicated immune system interactions. Vaccination against dengue can produce the same effect. This study estimates the average cost and disease burden for each case, in ten countries where these viruses spread, in order to understand how dengue vaccination and these immune interactions change the effects of an outbreak of both dengue and Zika. These estimates find that the immune interactions increase the direct impact of a dengue infection by a factor of 2–16 times, and the impact of a Zika infection by more than 100. Dengue vaccination increases the impact of a Zika case, has only a small effect on the impact of a dengue case when most people have already been infected by that type of dengue virus, and greatly reduces the impact of a dengue case when most people have been exposed to a different type of dengue virus than the one currently spreading.

## Introduction

Ever since the development of dengue vaccine candidates entered the trial stage, public health researchers have been estimating the potential impact of a dengue vaccine on transmission of the disease. Dengue, a flavivirus transmitted by *Aedes* mosquitoes, causes an estimated 100 million cases, and 40,000 deaths, per year [1], making it a major target of public health control in the over 100 countries where it is endemic. Some studies of dengue vaccination impact have taken into account the complex immunology entailed, where a person’s second dengue infection is much more likely to be severe (involving dengue hemorrhagic fever (DHF) or dengue shock syndrome) than the first. A phenomenon called antibody-dependent enhancement (ADE) may account for this severity: antibodies developed against the first dengue serotype encountered may recognize and bind to a second dengue serotype (there are four) without inactivating it, instead allowing the virus to hijack macrophages and replicate instead. Because the first licensed dengue vaccine, Dengvaxia, may also produce ADE in individuals without previous dengue exposure [2], the World Health Organization recommended its use only after screening positive for prior dengue exposure (seropositivity) [3], or (where such testing is unavailable) in settings where dengue prevalence is high and in individuals old enough that prior exposure is more or less certain.

However, recent studies suggest that a wider context may be needed in order to gauge the full impact of a dengue vaccination campaign in an endemic area, due to a broader impact of ADE. The Zika and chikungunya viruses are flaviviruses closely related to dengue, both genetically and physically. Some studies have identified ADE between these viruses and dengue (see [4] and references therein, as well as [5,6], though data on chikungunya remain scant as of this writing). In addition, mosquitoes which carry both dengue and Zika experience a related phenomenon: the viruses interact directly in the mosquito’s gut, in a way that increases the dengue viral load while lowering that for Zika [7]. A full understanding of the impact of dengue vaccination should thus take into account the possible effect of ADE on Zika infections, especially since the most severe Zika cases involve complications such as Guillain-Barré syndrome (GBS) and congenital Zika syndrome (CZS, associated with microcephaly).

Mathematical modeling studies provide a sophisticated way to estimate the interaction between disease transmission and control mechanisms, including vaccination’s indirect effects in preventing future cases in the unvaccinated. Indeed, researchers and vaccine developers have called for such studies to guide public health policies involving vaccination [8]. However, many of these works draw on parameter values used by other modeling studies, generating a long chain of citations in which the primary references, and the contexts in which estimates were generated, are lost. For example, a widely cited estimate of 0.67/day for the mean biting rate of an *Aedes* mosquito can be traced back through multiple citation chains to a 1956 study conducted in Kuala Lumpur, Malaysia [9] which stated, “*A. aegypti* is ready to feed twice or even more in the course of one gonotrophic cycle. This means that it may bite on two out of three days.” This summative description was not intended as an average over many careful measurements—but without a better primary estimate available, it has been used in a number of studies.

The severity of an outbreak involving multiple pathogens, such as dengue and Zika, may be measured in several ways. Since each virus has different consequences, complications, and durations, it may not be meaningful simply to sum the total number of infections of both types. Instead, measures must be developed which place the different types of infections in similar terms. Economic cost (measured in a given common currency at a given time) and disease burden (measured in years of life quality lost) are standard measures used to evaluate not only the impact of an outbreak itself, but that of any control measures brought to bear. Estimates of these quantities, which may be interrelated—for instance, a disease-related death causes loss of years of life as well as of economic earnings—are often used to justify and compare the large-scale application of specific controls.

The aim of this study is (first) to conduct a thorough review of parameters relevant to economic cost and disease burden calculations for dengue and Zika (including some related to transmission, screening, and vaccination), identify primary references, and (second) to develop a framework for estimating mean per-case cost and burden for dengue and Zika cases with and without ADE of disease, in order to build per-infection weighted averages over a population as functions of prior dengue seropositivity, prior and circulating dengue serotypes, screening method, and country. (Per-case estimates require no transmission dynamics model and are thus independent of whether ADE affects infectivity as well.) Trends in the results help evaluate the overall impact of dengue vaccination (and ADE more broadly) on a dual outbreak of the two viruses. Associating severe outcomes for each virus with ADE should produce an upper bound for the impact of ADE on a dual outbreak. Estimates focus on the ten countries where Phase III trials for Dengvaxia were held, in order to illustrate how cost and burden may vary by setting.

### Background: dengue infections and screening

Some background on transmission of the two viruses may help clarify why the estimates for each case type are broken down the way they are in this study. An individual’s first dengue infection is often mild and leads (after recovery) to long-lasting immunity against the given serotype, but after a short period of cross-immunity, the person remains susceptible to infection by other serotypes. Second infections (in which ADE is believed to play a role) are commonly more severe [10,11] (although any subsequent third or fourth infection by other serotypes are typically asymptomatic). Phase III trials of the CYD-TDV vaccine subsequently licensed as Dengvaxia revealed that it can act as a “silent infection” by priming dengue-seronegative individuals (with no prior dengue exposure) for ADE of any later dengue infection in the event of vaccine failure. Some controversy persists over whether a similar safety issue exists for the TAK-003 vaccine with some serotypes [12].

Screening for dengue seropositivity, where available, involves a tradeoff between accuracy and convenience. The World Health Organization identifies three broad categories of screening tests [3, Table 8]: plaque reduction neutralisation tests (PRNT), enzyme-linked immunoassay (ELISA), and rapid diagnostic tests (RDTs). PRNTs are considered the gold standard for detecting dengue seropositivity, but they are also so expensive, time-intensive (historically days long), and labor-intensive that they are impractical to use with a large-scale vaccination campaign. ELISA-based tests are faster (2–3 hours) and cheaper while still fairly accurate (sensitivity and specificity above 90%), but they are lab-based tests—necessitating separate visits for screening and vaccination, a major practical consideration in large-scale vaccination initiatives—and have been known to be cross-reactive with other flaviviruses including Zika, risking false positives. RDTs are by definition fast (within minutes) and inexpensive (relative to other tests as well as to vaccination itself), but existing RDTs have significant accuracy issues with either sensitivity or specificity, including cross-reactivity. This study therefore also develops separate estimates based on either ELISA or RDT screening.

## Methods

This study’s first aim involved a thorough review of the literature in order to identify primary references for basic quantities (referred to in this study as parameters) related to dengue and Zika transmission, screening and vaccination (for dengue), economic cost, and disease burden. This review cannot be classed as a single systematic review since different search terms were used for each quantity, and it is beyond the scope of a single study to document dozens of systematic reviews. However, for reference, search engines used included Google Scholar, PubMed, and Web of Science. Where multiple primary-source estimates were identified, they were pooled or averaged to propose a single value. For several parameters, direct estimates from primary literature were not available, so estimates were built from primary-source values for component quantities. Failing the availability of such values, estimates were extrapolated from the most closely related quantities available. In all cases, whether found or produced, the estimates yielded by this first aim are presented here as results (rather than as background or part of the methods).

The only variables (input quantities) for which fixed values were not adopted were country, dengue serotype (1–4), screener type (ELISA or RDT), and the proportion of the population (from 0 to 1) which seeks dengue [seropositivity] screening and vaccination. Parameter estimates obtained from the literature were typically global estimates except when available by serotype, country, and/or screener type. Of the ten Phase III trial countries, five are in Latin America (Brazil, Colombia, Honduras, Mexico, Puerto Rico), and five in southeast Asia (Indonesia, Malaysia, Philippines, Thailand, Vietnam). In this study, for simplicity we refer to Puerto Rico as a country. In the absence of cost data for the TAK-003 vaccine we use data on CYD-TDV to develop primary estimates. Estimates based on TAK-003 efficacy are tabulated in S1 Appendix and discussed in the Results section.

The study’s second aim required developing mathematical frameworks for estimating per-case economic cost and disease burden for dengue and Zika, as functions of the variables identified above. First-level estimates distinguish between cases with and without ADE of disease. Second-level estimates draw on observed prior dengue exposure levels (to infer proportions of ADE cases) and first-level estimates to produce overall per-case averages in the absence of vaccination. Finally, a set of estimates was produced for per-case cost and burden under the assumption of full compliance with a screen-and-vaccinate directive. Linear interpolation between estimates for no vaccination and universal screening for vaccination can then generate estimates for any desired level of compliance; in addition, the framework presented allows interested readers to generate estimates for any alternative parameter estimates. The underlying framework is presented here in Methods, with numerical estimates discussed in the Results section.

### Assumptions

All models are thought experiments, and the estimates developed in this study are based on assumptions and simplifications, many of which are not strictly accurate but allow us to make estimates. A list follows of the key assumptions used in developing results.

- In addition to the setting variables (country, dengue serotype, the type of screening test used, and compliance with screening-and-vaccination rollout), prior dengue seropositivity level and ADE characteristics affect measures of outbreak severity.
- Only one serotype of dengue is circulating in the population. Likewise, any prior dengue exposure in the population is limited to one serotype. However, near the beginning of an outbreak, per-case cost and burden estimates for alternative assumptions can be generated as weighted averages given a hypothesized frequency distribution of serotypes in circulation and/or prior exposure.
- ADE refers primarily to severity of disease, the clinical effects of infection on a person. In reviewing literature, we do address a study that implies ADE of dengue infectivity and consider its analogue for Zika, but the various per-case averages developed in this study are independent of any relative infectivity estimates.
- To investigate the impact of associating ADE with severe outcomes (cf. [13–15]), we assume that while some non-ADE dengue infections may require hospitalization, all ADE dengue cases require hospitalization, all fatal cases involve DHF (we do not distinguish DSS as a separate category), and all DHF cases are hospitalized ADE cases. We assume similarly that the more severe consequences of Zika infection are associated with ADE.
- Non-ADE dengue cases include self-managed, ambulatory, and hospitalized. ADE dengue cases include nonsevere hospitalized cases, nonfatal DHF cases, and fatalities. Non-ADE Zika cases are simple acute infections, while all instances of death, GBS, or CZS are associated with ADE Zika cases.
- For any given person, a tetravalent vaccine may “take” against one serotype but not against another; whether vaccination protects a given person from infection against one serotype is assumed independent of whether it protects that person against a different serotype. Further, vaccine protection is assumed to be “all or nothing” rather than “leaky”: that is, it provides a given individual either complete protection against all exposures to a given dengue serotype or none, rather than partial protection.

In addition, vaccine efficacy has been consistently reported to vary (often hugely) by dengue serotype (so we shall not consider this variation an assumption); this implies that average per-case cost and burden do so as well.

Note that this study’s focus on per-case estimates (rather than totals) requires no assumption about whether ADE, which amplifies severity (and therefore cost and burden) of a case, affects infectivity as well—nor indeed any assumptions about transmission dynamics at all, models for which will be left to other studies. We nevertheless review estimates for transmission-related parameters, for context and since some (like prior dengue seropositivity levels, and properties of vaccines and screeners) are relevant to per-case estimates.

Also, although the delay in reporting results for ELISA-based dengue screeners are strongly implied in the literature to decrease likely vaccination rates since some individuals might be unwilling or unable to make a repeat visit to the clinic for vaccination in the case of a positive screening result, we make here no assumption about such effects (since we make only per-case estimates).

Finally, detailing the equations used to generate per-case estimates at each level aims to facilitate further applications (thought experiments) by readers who wish to consider alternative values for some of the basic parameters. For example, a reader who wishes to consider a scenario where secondary dengue cases are half ADE, half subclinical could cut per-case cost and burden estimates for ADE dengue cases in half to estimate values for an average secondary case.

### Models of per-case economic cost and disease burden as functions of ADE

The primary quantities which this study aims to estimate are the average economic cost and disease burden for a single case of dengue or of Zika, as a function of dengue serotype, country, screening test, and the presence or absence of ADE, dengue vaccination, and prior dengue seropositivity. We build these quantities up from conceptually simpler quantities which we will refer to as parameters. The term *parameter* is commonly used in conjunction with a model, and the term *parameter estimation* in a biomathematical context often connotes the use of data fitting (calibration) to identify parameter values which make model outputs best match a dataset (often a time series). This study, however, focuses on deriving point estimates for relevant quantities based directly on primary literature. The model used here to derive the primary cost and burden estimates breaks each case type down into subtypes, assembles estimates for each subtype, and then produces a weighted average using the frequencies for each subtype.

We first build per-case estimates for ADE and non-ADE cases. We use *D*1 to tag non-ADE dengue cases, *D*2 for ADE dengue cases, and correspondingly *Z*1 and *Z*2 for Zika. Subtypes for dengue then include self-managed (*d*1*a*), ambulatory requiring medical attention (*d*1*b*), hospitalized non-ADE (*d*1*c*), nonsevere hospitalized ADE (*d*2*c*), nonfatal DHF (*d*2*d*), and fatalities (*d*2*e*). Subtypes for Zika include simple acute infection (*za*), GBS (*z*2*b*), CZS (*z*2*c*), and fatalities (*z*2*d*). For each subtype, values are developed from the literature to estimate the average per-case cost and burden as well as the proportion of non-ADE or ADE cases (as applicable) which are of that subtype (denoted *cost*, *burd*, *prop* respectively). The three *D*1 subtypes are mutually exclusive, as are the three *D*2 subtypes, so that


$$\begin{array}{c} prop\text{\_}d1a+prop\text{\_}d1b+prop\text{\_}d1c=1,\;prop\text{\_}d2c+prop\text{\_}d2d+prop\text{\_}d2e=1. \end{array}$$ (1)

However, the Zika case subtypes are not assumed mutually exclusive since simple acute infection is a baseline to which complications may be added.

The mean cost and mean burden per case as a function of case type (D1, D2, Z1, Z2) are then computed as averages (over the possible subtypes) weighted by the proportions of cases of each subtype. At the top level, this yields


$$\begin{array}{llll} cost\text{\_}d1 & =prop\text{\_}d1a\;*\;cost\text{\_}d1a+prop\text{\_}d1b\;*\;cost\text{\_}d1b+prop\text{\_}d1c\;*\;cost\text{\_}d1c,\; & & \;\\ cost\text{\_}d2 & =prop\text{\_}d2c\;*\;cost\text{\_}d2c+prop\text{\_}d2d\;*\;cost\text{\_}d2d+prop\text{\_}d2e\;*\;cost\text{\_}d2e,\;\\ cost\text{\_}z2 & =cost\text{\_}za+prop\text{\_}z2b\;*\;cost\text{\_}z2b+prop\text{\_}z2c\;*\;cost\text{\_}z2c+prop\text{\_}z2d\;*\;cost\text{\_}z2d.\; & & \; \end{array}$$ (2)

The costs and burdens for non-ADE Zika (Z1) cases, as well as screenings and vaccinations, are simpler as they have no subtypes. Estimating the mean disease burden per case type for D1, D2, and Z2 involves computations exactly analogous to those for mean cost per case given above. The definition of disease burden, as proposed by Murray [16], is measured in disability-adjusted life years (DALYs), consisting of years of life lost (YLLs) due to premature death and years of healthy life lost due to disability (YLDs), the latter computed using disability weights (DW) estimating how much of the quality of life is lost during a given illness.

Estimates for a few subtypes require further decomposition because of the nature of the data available. First, the per-case economic costs for hospitalized non-DHF cases (*cost*_*d*2*c*) and [hospitalized] nonfatal DHF cases (*cost*_*d*2*d*) must be written in terms of three quantities estimated from the literature: the average cost of a nonfatal hospitalized dengue case, say $c_{H}$, the proportion of hospitalized dengue cases involving DHF, say *h*, and the ratio of cost per nonfatal DHF case to cost per hospitalized non-DHF dengue case, say *k*. From the equations


$$\begin{array}{c} k=\frac{cost\text{\_}d2d}{cost\text{\_}d2c},\;c_{H}=(1-h)\;*\;cost\text{\_}d2c+h\;*\;cost\text{\_}d2d,\; \end{array}$$ (3)

some algebra shows that


$$\begin{array}{c} cost\text{\_}d2c=\frac{c_{H}}{1+h(k-1)},\;cost\text{\_}d2d=\frac{c_{H}k}{1+h(k-1)}. \end{array}$$ (4)

Second, the cost per simple acute Zika case was estimated using estimates (assumed global) of the proportion of Zika cases with symptoms (*prop_symp*), the proportion of cases for which medical care is sought (*prop_seek*), and the average duration of symptoms in days (*dur_symp*), and data from each country on the cost of a single outpatient visit (*cost_care*) and the gross national income (GNI, previously called GNP per capita) as follows:


$$\begin{array}{c} cost\text{\_}z2a=prop\text{\_}symp\;*\;(prop\text{\_}seek\;*\;cost\text{\_}care+dur\text{\_}symp\;*\;GNI∕365) \end{array}$$ (5)

Third, although a UN report estimated costs per case for the Zika complications GBS and CZS in the five Latin American countries [17, Tables 5A, 6A] (in 2015USD), it calculated costs only for CZS cases in which the patient’s child survived. This study therefore adjusts those figures to take into account the estimated 30% of CZS pregnancies that lead to early pregnancy loss or perinatal death [18,19]. Specifically, we estimate the cost by multiplying the figure from the UN report by 70%, and adding 30% times the estimated lifespan productivity for the given country to incorporate the lost productivity for the lives lost altogether due to CZS. Lifespan productivity for each country was estimated by multiplying the 2015 average annual household per capita income by the life expectancy using 3% annual discounting (results were then brought forward to 2024USD).

Next, because cost data for the two Zika complications were unavailable for the five study countries in southeast Asia, these values were estimated using the patterns across countries and the patterns across cost types in the existing estimates. In particular, by treating the estimated costs of the different dengue and Zika case types (see “Economic cost parameters” in the Results section) as a matrix, approximating the unknown Zika costs becomes a matrix completion problem, to which the technique known as CUR decomposition [20] can be applied. This technique treats the known complete rows as a basis *R* for the row space of the full matrix and the known complete columns as a basis *C* for the column space of the complete matrix, and writes the full matrix in terms of *R*, *C*, and their intersection *U*.

In this case, since equation (4) makes *cost_d2d* a multiple of *cost_d2c*, we can use the other four dengue cost components as a basis *R* for the row space. Using the full set of dengue and Zika cost components for the five Latin American countries as a basis for the column space, the resulting CUR decomposition showed that initial estimates for costs per GBS and CZS case in Honduras generated from the UN report estimates were inconsistent with the rest of the data (in particular they were too high). We therefore use the columns for the other four Latin American countries as a basis for the column space and generate estimates for GBS and CZS case costs in Honduras and the five southeast Asian countries using a rank-4 CUR decomposition.

Finally, the burden per ZIKV GBS case includes a component for the GBS case proper as well as a component for associated deaths, which depends on the case fatality ratio (CFR) and the expected years of life lost (YLLs) per death, which is the difference between the expected lifespan (say *L*) and the expected age at death:


$$\begin{array}{c} burd\text{\_}z2b=burd\text{\_}GBS+CFR\text{\_}GBS^{*}(L-age\text{\_}death). \end{array}$$ (6)

Meanwhile, following Puntasecca *et al.* [19], the burden of CZS includes three components: loss to the child, YLDs of the mother due to depression (*burd_depr*), and YLDs for the child’s caretaker in case of survival (*burd_ct*). Loss to the child is the entire lifespan in case of death from CZS, and lifelong ($L_{CZS}$) severe disability (with corresponding disability weight (DW)) in case of survival. This yields


$$\begin{array}{c} burd\text{\_}z2c=(L-(1-CFR_{CZS})(1-DW_{CZS})L_{CZS})+burd\text{\_}depr+burd\text{\_}ct. \end{array}$$ (7)

We observe that *burd_z2c* (and consequently *burd_z2*) increases as (when) $L_{CZS}$ decreases from *L*, since a reduced lifespan corresponds to more years lost to CZS.

### Models of per-case economic cost and disease burden as functions of dengue screening, vaccination, and prior exposure

Specific scenarios involving prior dengue exposure and/or screen-and-vaccinate campaigns generate their own per-case estimates for cost and burden by identifying the proportions of the population who would develop ADE or non-ADE cases of each virus (or, for those protected against the circulating dengue strain, who would develop no infection at all). In each scenario, the estimated per-exposure average *per_exp* (substituting *cost* or *burd* for *value*, *d2* or *z2* for *ADE*, and *d1* or *z1* for *nonADE*) takes the general form


$$\begin{array}{c} per\text{\_}exp=prop\text{\_}ADE\times value\text{\_}ADE+prop\text{\_}nonADE\times value\text{\_}nonADE. \end{array}$$ (8)

However, note that in cases where vaccination or prior exposure prevents some dengue cases, the per-infection value *per_inf * for dengue is not the same as the per-exposure value (rather, it is greater), since not all exposures lead to cases, and a per-infection value is an average over only those exposures which do lead to cases. In such cases, we must rescale by the proportion of exposures which lead to infection, so that


$$\begin{array}{c} per\text{\_}inf=per\text{\_}exp∕(prop\text{\_}ADE+prop\text{\_}nonADE). \end{array}$$ (9)

By taking into account the background level of dengue seropositivity documented in each region by [21] and [22], one can develop a population-level weighted average per-infection cost and burden for each virus at the outset of an outbreak (in the absence of vaccination), using the per-case estimates for ADE cases and non-ADE cases. If we denote the dengue seropositivity level as *α* (0 ≤ *α* < 1), and assume that prior seropositivity triggers ADE in any subsequent infection, then (8) and (9) give the same value, a per-case average of *α* times the per-ADE-case value plus  ( 1 - *α* )  times the per-non-ADE-case value. The exception is when the dengue serotype currently circulating is the same as that of any prior exposure: in this scenario, prior exposure leads (we assume) to lifelong immunity against reinfection by that same serotype, so the weighted per-exposure average for dengue is then instead given (via (8)) by *α* × 0 + ( 1 - *α* ) × *value*_*nonADE*, while the per-infection value (i.e., conditioned on successful infection) remains the per-non-ADE-case value. (For simplicity we ignore here scenarios where multiple dengue serotypes currently circulate and some but not all match those of prior exposure—but see the note on such scenarios below.) More generally, note that as *α* varies from 0 to 1, the per-infection cost and burden vary linearly from the per-case non-ADE value to either the ADE value (for Zika and for dengue with different serotypes) or 0 (for dengue with the same serotype).

Estimating per-infection cost and burden under a dengue vaccination campaign introduces a number of new quantities to the calculation, namely the properties of the screening test (sensitivity and specificity) and vaccine (serotype-specific efficacy) used, as well as the proportion of the population seeking vaccination (which we assume independent of prior dengue exposure, as individuals often do not know their serostatus). Vaccine efficacies are also used to compute the proportions of vaccinations which result in different outcomes, such as tetravalent failure, threefold failure (protection against only one serotype), etc. The resulting estimates, which require more complex weighted averages, then vary by not only country but screening test (ELISA or RDT) and dengue serotype (1 through 4), as well as depending on prior exposure and the proportion of people seeking vaccination.

We introduce notation to denote these quantities, summarized in Table 1. The proportions denoted *a* or *b* with various subscripts are defined as follows: Fourfold vaccine failure is the product of proportions of failure (non-efficacy) in each serotype, $a_{0}=\underset{i}{\prod }(1\;-\;\eta_{i})$. Success for only one, noncirculating serotype is the sum of the disjoint proportions in which each noncirculating serotype (in turn) succeeds but the other three fail, $a_{\omega }=\underset{k\neq j}{\sum }\eta_{k}\underset{i\neq k}{\prod }(1\;-\;\eta_{i})$, where the circulating dengue serotype is denoted *j*. Success for either the circulating serotype or any two or more serotypes (which we assume to cause effective immunity against further symptomatic dengue infections of any serotype) is then the complement of the two previous proportions, $1\;-\;a_{0}\;-\;a_{\omega }$. Meanwhile, if we denote by *n* the dengue serotype of prior exposure, then the proportion of dengue-seropositives who receive no new protection through vaccination is just the product of non-efficacies in the other three serotypes, $b_{1}=\underset{i\neq n}{\prod }(1-\eta_{i})$, while those who do receive new protection is its complement, $b_{2}=1-b_{1}$. Note that, of these compound proportions, $a_{0}$ is independent of which dengue serotypes are circulating or in individuals’ infection histories, $a_{\omega }$ is specific only to the currently circulating serotype, and $b_{1}$ and $b_{2}$ are specific only to the serotype of prior exposure.

**Table 1:** Notation used in expressing weighted-average per-infection estimates under tetravalent vaccination.

Notation	Definition
*α*	Proportion of the population with prior dengue exposure (seropositivity)
*p*	Proportion of the population seeking screening
*ψ*	Screening test sensitivity (probability of a positive result, given a positive sample)
*χ*	Screening test specificity (probability of a negative result, given a negative sample)
$\eta_{i}$	Vaccine efficacy (proportion of infections prevented) against serotype *i*
$a_{0}$	Proportion of vaccinations leading to fourfold (total) vaccine failure
$a_{\omega }$	Proportion of vaccinations leading to protection against only one, noncirculating serotype
$b_{1}$	Proportion of vaccinated dengue-seropositives who receive no new protection through vaccination
$b_{2}$	Proportion of vaccinated dengue-seropositives who receive protection against at least one “new” serotype
$c_{H}$	average cost of a nonfatal hospitalized dengue case
*h*	proportion of hospitalized dengue cases involving DHF
*k*	ratio of cost per nonfatal DHF case to cost per hospitalized non-DHF case
*L*	expected lifespan

We thus compute population-level weighted average cost and burden per infection, assuming a single screening/seeking. First, independently of infection, we compute an average per-person cost for screening and vaccination. A proportion *p* of individuals seek (and thus pay for) screening. (We note *p* cannot actually reach 1 since not everyone is old enough to be eligible.) Of those, a proportion *αψ* both have prior exposure and test positive, and a proportion  ( 1 - *α* ) ( 1 - *χ* )  are seronegative but test positive anyway (due to false positive screening); thus a proportion *p* ( *αψ* + ( 1 - *α* ) ( 1 - *χ* ) )  of individuals pay for vaccination as well.

In a scenario where the circulating and historical serotype of dengue are the same, a proportion *α* of the population are immune to it and involve no cost if exposed to dengue. Of the 1 - *α* seronegatives, a fraction *p* ( 1 - *χ* )  seek vaccination, screen false positive, and receive the vaccine. Of those, a proportion $a_{0}$ receive no protection and develop non-ADE cases if exposed, while a proportion $a_{\omega }$ (serotype-dependent) receive protection against only one, noncirculating serotype and thus develop ADE cases if exposed to the circulating serotype. The remainder of the false-positive vaccinated receive effective protection against the circulating serotype and develop no symptoms (hence no cost) if exposed. Meanwhile, the remainder of the seronegatives either do not seek vaccination or else correctly screen seronegative, and develop non-ADE cases if exposed. The proportion of the population which develop non-ADE cases is thus


$$\begin{array}{c} (1-\alpha )p(1-\chi )a_{0}+(1-\alpha )(1-p(1-\chi ))=(1-\alpha )(1-p(1-\chi )(1-a_{0})), \end{array}$$ (10)

and the overall mean cost (or burden) per exposure for dengue is


$$\begin{array}{c} (1-\alpha )p(1-\chi )a_{\omega }\times value\text{\_}ADE+(1-\alpha )(1-p(1-\chi )(1-a_{0}))\times value\text{\_}nonADE. \end{array}$$ (11)

The corresponding per-infection average requires rescaling (11) by the sum of the two proportions, $\left(1-\alpha )[1-p(1-\chi )(1-a_{0}-a_{\omega })\right]$.

In a scenario where instead the circulating and historical serotypes of dengue differ, of the seropositives *α*, a proportion $p\psi b_{2}$ seek vaccination, correctly screen seropositive and receive protection which prevents symptomatic infection, thus incurring no costs if exposed. Any other outcome for seropositives (vaccine failure or no vaccination) results in no protection against the circulating serotype, and thus an ADE case upon exposure to it. The effect on dengue-seronegatives 1 - *α*, meanwhile, is identical to that described above in a scenario where the circulating and historical serotype are the same, since seronegatives have no exposure to the historical serotype. The overall mean cost or burden per exposure for dengue is then


$$\begin{array}{llll} \left[\alpha (1-p\psi b_{2})+(1-\alpha )p(1-\chi )a_{\omega }\right] & \times value\text{\_}ADE+(1-\alpha )(1-p(1-\chi )(1-a_{0}))\; & & \;\\ & \times value\text{\_}nonADE.\; & \end{array}$$ (12)

In a setting where the population has a high level of prior dengue exposure and any element of a vaccination campaign (compliance *p*, screening sensitivity *ψ*, vaccine efficacy $b_{2}$) is limited, the additional term $\alpha (1\;-\;p\psi b_{2})$ times the high impact of an ADE case will make the average impact of a dengue infection much higher if the circulating and historical serotypes of dengue differ—as was notably the case in Cuba in 1981, when a DENV-2 outbreak caused over 10,000 cases of dengue hemorrhagic fever just four years after a DENV-1 outbreak—than if they match.

The corresponding per-infection average (9) rescales (12) by the proportion $\alpha (1-p\psi b_{2})$  +  $\left(1-\alpha )[1-p(1-\chi )(1-a_{0}-a_{\omega })\right]$ of individuals susceptible to infection.

Regardless of the circulating and historical serotypes of dengue, all dengue-seropositives, whether by prior exposure *α* or by vaccination (with at least one “take”) following screening failure $\left(1-\alpha )p(1-\chi )(1-a_{0}\right)$, develop ADE cases if infected with Zika, while the rest develop non-ADE cases. Thus the mean cost or burden per case (exposure or infection) for Zika is


$$\begin{array}{llll} \left[\alpha +(1-\alpha )p(1-\chi )(1-a_{0})\right] & \times value\text{\_}ADE+(1-\alpha )(1-p(1-\chi )(1-a_{0}))\; & & \;\\ & \times value\text{\_}nonADE.\; & \end{array}$$ (13)

### Scenarios involving multiple prior and/or circulating dengue serotypes

For scenarios where multiple dengue serotypes are involved in prior exposure and/or in the current outbreak, per-exposure cost or burden at the beginning of an outbreak can be estimated by taking a weighted average over every possible combination of prior and present serotypes, where the weights are the proportions of either prior exposure or new infections due to the given respective serotypes. An example is given at the end of the Results section.

## Results

### Parameter estimation

Wherever possible, estimates were made country-specific; this was generally easier to do for dengue than for Zika, since dengue has been a perennial threat in these countries for much longer and studied in greater depth. Estimates also used primary references whenever possible.

### Transmission-related parameters

Estimated values for transmission parameters are summarized in Table 2. Although transmission characteristics certainly vary by region (and time of year), primary data for estimates are so scarce that in most cases we use global annual means here. Values are reported by country, DENV serotype, or screener when corresponding data are available, regardless of whether a broad consensus exists that these setting variables cause innate differences. Values in the table taken directly from primary sources are followed by a citation; others are estimated below as composite values.

**Table 2:** Transmission-related parameters for dengue and Zika, and their estimated values. All values not identified by source were estimated in this paper; see the first part of the results section for details. * first 25 months, ages 9–16.

Definition	Value(s)
biting rate for *Aedes aegypti* mosquitoes: engorged females	0.76/day (Thailand), 0.63/day (Puerto Rico) [24]
all females on humans	0.41/day (Thailand), 0.35/day (Puerto Rico) [23]
human-to-mosquito dengue transmission probability	1 (Brazil [25]), 0.9185 (Thailand [26])
mosquito-to-human dengue transmission probability	0.6 (Brazil [25]), 0.710 (Thailand [26])
human-to-mosquito Zika transmission probability	0.70
mosquito-to-human Zika transmission probability	0.50
dengue recovery rate, by serotype	1/(4.2d,4.2d,7.2d,1.5d) [28]
Zika recovery rate	1/wk
relative host dengue infectivity due to ADE, by serotype	1.4, 1.1, 1.5, 1 [33]
relative host Zika infectivity due to ADE	2.14
relative dengue infectivity of coinfected mosquitoes	12
relative Zika infectivity of coinfected mosquitoes	0.17
human population size (millions), by country	Bra 217, Col 49.1, Hon 9.46, Mex 129, PR 3.10,
	Ind 277, Mal 33.9, Phi 115, Tha 69.6, Vie 104
mosquito/human ratio	0.52
average human lifespan, by country (yrs)	Bra 75.44, Col 78.10, Hon 73.40, Mex 76.47, PR 81.47,
	Ind 72.06, Mal 75.28, Phi 70.36, Tha 77.55, Vie 74.47
mean female adult mosquito lifespan	28 days = 0.0755 yrs
dengue vaccine eligibility age	9 yrs
background dengue seropositivity	0.749 (Latin America) [22], 0.718 (SE Asia) [21]
screening sensitivity	0.876(ELISA), 0.635(RDT)
screening specificity	0.975(ELISA), 0.987(RDT)
vaccine efficacy, by dengue serotype (1–4)	0.503, 0.423, 0.740, 0.777 (CYD-TDV, *, Latin America) [22, Table 3]
	0.500, 0.350, 0.784, 0.753 (CYD-TDV, *, SE Asia) [21, Table 3]
	0.737, 0.977, 0.626, 0.632 (TAK-003, first year) [47, Table 2]

A mechanistic approach to estimating infection rates is to multiply mosquito biting rate by the probability of the given transmission type. No primary sources were found measuring biting rate in bites per mosquito per day, though a few measured bites per gonotrophic cycle (without giving cycle duration). One 1956 study in Kuala Lumpur, Malaysia estimated a mean of 2 bites per gonotrophic cycle, with a gonotrophic cycle of about 3 days, which has been widely cited for yielding a biting rate of 0.67/day [9]. More recently, a systematic review, which also recognized the need to distinguish biting rate per day from bites per gonotrophic cycle and duration of gonotrophic cycle, found so-called multiple feeding studies to be most helpful in developing biting rate estimates [23]. The paper reviewed the methodology of such a study estimating biting rates for engorged female *Ae. aegypti* mosquitoes in Thailand and Puerto Rico of 0.76/day and 0.63/day, respectively [24]. The authors of the review argued for adjustments in estimating the mean biting rates for all (not just engorged) female *Ae. aegypti* mosquitoes which result in lower averages of 0.41/day and 0.35/day, respectively.

Two primary sources were found which estimate the transmission probability of dengue from mosquito to human: 0.6 in Brazil in 2010 [25] while in Thailand in 2011, 0.675 for DENV-1 and 0.745 for DENV-2 (averaging 0.710) [26]. For dengue transmission from human to mosquito, the same two sources give 1 and 0.934, 0.903 (averaging 0.9185), respectively.

For Zika there are even fewer data on transmission than for dengue; in particular the only primary source found for transmission probabilities was Tesla et al. [27], which measured vectorial capacity in mosquitoes as a function of viral concentration. They found that the probabilities of *Aedes aegypti* acquiring ZIKV in its midgut and then of transmitting it in its saliva dropped off sharply below $10^{4.98}$ RNO copies per mL, and that the product of the two probabilities reached 0.2 at a concentration of $10^{5}$/mL and 0.35 for $10^{6}$/mL. Since in the field *Ae. aegypti* are competent vectors we can take the latter value for the product, and assume that the ratio between the two probabilities (host to vector and vice versa) is the same as for dengue, namely that the mosquito’s acquisition probability is 0 . 946 ∕ 0 . 673 = 1 . 4 times as great as the mosquito’s transmission probability (the averages of the three dengue one-way transmission probabilities given above are 0.946 human to mosquito and 0.673 vice versa). From these two conditions we deduce *prob_mhz*=0.50 and *prob_hmz*=0.70. (It is worth noting that, in places where $R_{0}$ has been estimated for both viruses directly from epidemiological data, dengue and Zika have similar values, with dengue’s sometimes slightly higher, which is consistent with the values produced by these parameters.)

Literature commonly cites recovery periods for dengue of about 1 week, varying from 2 to 9 days. These are typically secondary sources which cite a chain of references which, if traced back, fail to lead to a primary source with the given duration of infection. The only primary source we found for this was the study of Anderson et al. [28], who gave data specific to DENV serotype. Although their study focused on Thai children aged 5–15, and their data may therefore not generalize to patients of all ages in all countries, their estimates fall within the range commonly given, so in the absence of further primary data we report them.

Likewise many studies state a recovery period for Zika of about 1 week (e.g., [29]), but primary sources were difficult to find. One study of [already] Zika-positive individuals reported, “The maximum number of days during which ZIKV RNA was detected was 7 days post-symptom onset in saliva and serum/plasma” [30], significant since duration of infectivity to mosquitoes is based on virus presence in the blood. One literature review stated (without further detail) that symptoms resolve within 2 weeks [31], while the European Union web site Zika factsheet states that its duration is 2–7 days without severe complications [32]. We use the consensus figure of 1 week to estimate the recovery rate.

There is no consensus on whether ADE affects infectivity, but we report here one study which examined the relative dengue infectivity of ADE cases. Chikaki and Ishikawa inferred relative infectivity by DENV serotype due to ADE (as part of the probability of dengue transmission from mosquito to human per bite) from data, using the DENV-4 probability as baseline [33], so we use those values (all between 1 and 1.4) for serotype-specific infectivity amplification factors. It should be noted, however, that some studies (either *in vitro* or else *in vivo* in mice and macaques) have found differences in dengue viral load of orders of magnitude between infections with and without ADE, so the true multipliers for infectivity may be much higher. George et al. found that Zika antibodies caused ADE of DENV-2 infections in macaques of a magnitude similar to that caused by antibodies to DENV-1, 3, or 4 [34], so we assume the same values for the amplification factors regardless of ADE cause.

A more direct connection between viral load and infectivity must be considered in order to estimate a potential relative Zika infectivity for ADE cases, under an ADE-of-infection hypothesis. One review found that DENV antibodies increased Zika viral loads *in vitro* by a factor of “over 100” in several studies [35]. One such study reported factors of 60–248 [36]; another reported factors of 140, 275, and 200 [37]. We take 140 (the average of factors given in the first study, and one of those in the second, supported by Fig 6a in [37]) as a typical amplification factor, and make an additional assumption that actual infectivity increases logarithmically with viral load, as George et al. reported a viral load increase of $10^{1.5}$ for dengue [34], with 1.5 close to Chikaki and Ishikawa’s estimated relative infectivity values for ADE-magnified DENV. Thus we compute  log ⁡ 10140=2.14.

Chaves et al. compared viral loads in monoinfected and coinfected *Aedes aegypti* mosquitoes for both DENV and ZIKV [7]. Specifically, in samples from mosquito salivary glands (relevant since this is the mechanism of transmission to hosts) they found $1.7\;\times \;10^{4}$ DENV cDNA copies in DENV-monoinfected mosquitoes and $2.1\;\times \;10^{5}$ in coinfected mosquitoes, a 12-fold increase. Meanwhile they found $8.8\;\times \;10^{6}$ ZIKV cDNA copies in ZIKV-monoinfected mosquitoes’ salivary glands vs. $1.5\;\times \;10^{6}$ in coinfected mosquitoes, 0.17 times as much. These two factors can act as proxies for relative dengue and Zika infectivities, respectively, of coinfected mosquitoes. A more recent study found similar results as well as an overall increased infection rate for coexposed *Ae. aegypti* mosquitoes relative to monoexposed ones [38].

Human and mosquito population sizes are relevant to disease transmission in two ways: first, since infectious contacts are generally agreed to be driven by mosquito density, the ratio of mosquitoes per human, say $N_{m}∕N_{h}$, affects the transmission rate (cf. the standard Ross-Macdonald approach), and second, the human population size scales total costs and disease burdens in each country. Although it is unreasonable to assume homogeneous mixing of hosts and vectors at the level of an entire country, given a fixed ratio $N_{m}∕N_{h}$ the predicted prevalence of each disease (i.e., per person) over time is independent of scale. We therefore estimate the vector-host ratio separately, and suggest using the total human population of each country (values listed are taken from the CIA Factbook [39] for 2022) only for studies aiming to estimate total cost and disease burden. In the literature, although there are many studies of the effects of variables such as temperature on mosquito population density, only two were found which gave estimates of absolute mean population density, both from Brazil, and their resulting ratios $N_{m}∕N_{h}$ are close enough to use the average. Rodrigues et al. estimated 0.42 mosquitoes per person [40]; Maciel-de-Freitas et al. generated data from which a figure of 0.62 mosquitoes per person can be calculated (8333 mosquitoes in 0.79 sq.km. vs 62509 people in 3.7 sq.km.) [41]. We ignore seasonal effects and take the figure as annual mean.

To estimate human mortality we take the average of the expected lifespans for each country from the WHO Global Health Observatory [42] and the CIA Factbook [39] (which were mostly but not always within 1 year of each other), except for Puerto Rico, for which the WHO GHO does not provide a separate estimate.

The adult mosquito lifespan is widely taken in the literature as 2 weeks for any species, but without reference to any primary source confirming the data. Tesla et al. found an adult lifespan of 2–6 weeks for *Aedes aegypti*, varying by temperature within a range of roughly 15–35∘C [43]. Another study by the same authors found a mean lifespan of 27.5 days post feeding [27]. Lambrechts et al. found an average adult lifespan of 27.56 days in a Thailand laboratory, but also found that the amplitude of daily temperature swings (diurnal temperature range, DTR) had an important effect on both lifespan and mosquito acquisition of dengue per bite [26]. Given the complexity of their models, we suggest this mean value, which matches the data from [27] and falls squarely in the middle of the range given by [43], while noting that it is twice the more commonly assumed 14-day lifespan, which undoubtedly affects disease transmission (in particular, it increases it since mosquitoes remain carriers for their entire lives).

Prior dengue seropositivity estimates come from the two Phase III clinical trial studies for CYD-TDV/Dengvaxia. In Latin America, Villar et al. found an overall rate of 0.749 for all dengue serotypes combined among children ages 9–11 [22]. Likewise Capeding et al. found an overall rate of 0.718 for all dengue serotypes combined among children ages 6–11 in southeast Asia [21]. Note that it is likely higher for adults.

The sensitivity and specificity of dengue seropositivity screening tests vary significantly by test. We distinguish values for each of the three types of test described in the introduction, while recognizing that each of the latter two categories comprises many different tests using different criteria. The PRNT test, the gold standard for testing, is often considered to have full (100%) sensitivity and specificity, although its cost and timeframe make it prohibitively expensive and slow to use in the kind of large-scale vaccination effort envisioned in this study. ELISA tests, which generally require at least 2 hours to develop results, may use any of three different criteria, all of which have different properties: NS1 antigen or IgG or IgM antibodies. RDTs may also use these criteria but a different process. The WHO gave estimates for sensitivity and specificity of NS1 ELISA and IgG ELISA tests [3], and Lee et al. tested 28 different combinations of criteria, test types, and manufacturers [44]. They found the two NS1 ELISA tests to rank highest (100% sensitivity, 99.2% specificity, and no cross-reactivity to chikungunya virus) but noted a very low sample size for that test compared to the other tests. In general they found that RDTs had the highest specificity but markedly lower sensitivity than ELISA tests. We use for this study the average sensitivity and specificity values for their single-criterion ELISA and RDT test results (they also gave results for tests using multiple criteria—NS1, IgG, IgM—but this effectively uses multiple tests and would thus cost more). As a side note, Lee et al. reported a range of 30–90% cross-reactivity to chikungunya for the IgG-based tests [44], in line with the 54% figure of van Meer et al. [45] cited by the WHO [3].

Serotype-specific vaccine efficacy estimates for Dengvaxia come from the same two clinical trial studies: [21,22], using values for children in the Latin American or southeast Asian countries, respectively, and from a retrospective study covering both regions [46] (serostatus-specific). Global values for the TAK-003 vaccine come from [47] for the first year and from [48] over the first 4.5 years, by serostatus. Since the serostatus-specific values include some negative efficacies, which do not lead to meaningful estimates, we use the estimates from the earlier studies, while noting that the lower efficacies reported by the later studies for each vaccine indicate some waning of protection over time. [For convenience, vaccination outcome parameters $a_{0},a_{\omega },b_{2}$ defined in the Methods section, which are functions of serotype-specific efficacies, are calculated for the data from the three earlier studies and listed in Table A in S1 Appendix].

**Table 3:** Parameters representing proportions of each case type (d1, d2, z2) with various characteristics or outcomes. Self-managed cases include asymptomatic; ambulatory cases seek treatment; severe nonfatal involve DHF/DSS. See the corresponding results subsection for further detail.

Parm.	Prop. of cases that are...	Value in...									
		Brazil	Colo.	Hond.	Mexico	P.R.	Indo.	Mala.	Phil.	Thai.	Viet.
prop_d1a	...self-managed	21.33%	27.39%	32.86%	28.36%	33.28%	27.99%	33.70%	49.93%	29.99%	8.15%
prop_d1b	...ambulatory	77.87%	71.68%	66.54%	69.02%	56.61%	60.86%	56.11%	47.51%	64.39%	87.17%
prop_d1c	...hospitalized	0.80%	0.93%	0.59%	2.62%	10.11%	11.15%	10.19%	2.56%	5.62%	4.69%
prop_d2c	...hospitalized	4.72%	4.72%	4.72%	4.72%	4.72%	4.72%	4.72%	4.72%	4.72%	4.72%
prop_d2d	...severe nonfatal	94.52%	94.41%	94.31%	95.08%	95.18%	95.17%	95.21%	94.83%	95.18%	95.25%
prop_d2e	...fatal	0.75%	0.86%	0.97%	0.19%	0.09%	0.10%	0.06%	0.45%	0.10%	0.03%
prop_z2b	...GBS/chronic	0.028%									
prop_z2c	...CZS	0.64%									
prop_z2d	...fatal	0.0032%									

### Proportions of case types

Proportions of dengue and Zika cases with different characteristics, needed in order to estimate overall per-case cost and burden, are presented in Table 3 and discussed below.

The proportions for dengue are based primarily on the extensive study of [49], which in Table A3 of its appendix breaks down data for each country on dengue cases in the categories nonmedical (self-managed), ambulatory, hospitalized nonfatal, and fatal.

To subdivide hospitalized cases between ADE and non-ADE, we pool four studies in which the proportion of hospitalized cases involving primary dengue infections varied from 6.8% to 34.4% [11], [50], [51], [52]: 26/379, 306/1064, 18/78, and 37/108, for an overall proportion of 387/1629 or 23.8% (the remaining 76.2% being secondary). (Averaging the four studies’ rates would give a similar figure of 23.2%.) For this particular proportion we use primary and secondary as proxies for non-ADE and ADE.

Meanwhile, we estimate the proportion of severe (DHF) cases by averaging the proportions of hospitalized cases involving DHF in [53,54] (58.2% and 87%, respectively) to get 72.6%. Nonsevere hospitalized cases then account for the difference between the 76.2% of hospitalized cases that are assumed ADE infections and the 72.6% of hospitalized cases that are DHF, thus 3.6%, making *prop*_*d*2*c* = 0 . 036 ∕ 0 . 762 = 0 . 0472, applied for all countries. The proportion of ADE cases that are fatal (*prop_d2e*) is then the number of fatal cases divided by the 76.2% of hospitalized cases (nonfatal or fatal) which are assumed ADE infections, and the remainder (*prop*_*d*2*d*, via (1)) are nonfatal DHF cases.

An extensive literature review turned up no estimates of the proportion of Zika cases involving ADE, so as a crude approximation we use the estimated dengue seropositivity level, to rescale (divide) the overall [global] Zika complication proportions reported by Puntasecca *et al.* [19].

### Economic cost parameters

Table 4 summarizes cost estimates for each case subtype. Except as noted, all costs include direct costs, as well as indirect costs (productivity losses) to the patient and family (caregivers), and are assumed to be incurred at the time of infection (but with annual discounting for future productivity lost). All costs are brought forward to 2024USD from source data using a 3% annual discount rate.

**Table 4:** Parameters representing subtype-level average costs (in 2024 USD) for each case type (d1, d2, z2) with various characteristics or outcomes. Self-managed cases include asymptomatic; ambulatory cases seek treatment; severe nonfatal involve DHF/DSS. See Results section for further detail.

Parm.	Cost of cases that are...	Value in...									
		Brazil	Colo.	Hond.	Mexico	P.R.	Indo.	Mala.	Phil.	Thai.	Viet.
cost_d1a	...self-managed	395.89	143.96	37.37	161.96	1295.64	58.14	265.77	48.45	30.45	20.76
cost_d1b	...ambulatory	465.10	264.39	78.90	632.59	1863.18	117.66	614.60	202.10	231.17	45.68
cost_d*c	...hospitalized	888.97	888.97	300.13	1536.01	6601.64	439.35	1037.32	701.82	763.44	91.29
cost_d2d	...severe nonfatal	1149.81	1149.81	388.19	1986.70	8538.68	568.26	1341.69	907.74	987.45	118.08
cost_d2e	...fatal	303,701	249,616	71,846	286,000	804,027	107,423	309,402	98,531	204,079	60,960
cost_za	...acute	20.39	16.56	6.88	28.70	60.60	11.15	27.60	9.74	16.56	9.51
cost_z2b	...GBS/chronic	290,296	229,149	62,729	272,306	427,898	94,315	329,503	75,039	199,796	67,095
cost_z2c	...CZS	869,225	659,296	178,145	769,772	1,167,724	268,500	968,294	202,002	565,521	196,923
cost_z2d	...fatal	same as cost_d2e (see main text )									
cost_scr	screening	18.14	17.22	15.79	18.14	24.01	16.31	18.40	16.05	17.22	11.71
cost_vac	vaccination	229.98	146.55	121.19	164.28	267.16	138.16	166.40	134.21	144.08	118.84

Costs per dengue case draw on data given in Shepard et al. [49], where Table A4 gives mean cost per case for self-managed (“non-medical”), ambulatory, nonfatal hospitalized, child fatal, and adult fatal cases, in 2013USD ([49] breaks the cost into direct and indirect, but here we combine them). Using the data on numbers (and thus proportions) of child vs. adult fatal dengue cases by country in their Table A3, a mean cost per overall fatal case can be calculated for each country as a weighted average.

Since hospitalized nonfatal cases include both non-severe and severe (DHF) cases, in order to derive separate costs for each category we look at other studies which distinguish costs for DHF cases vs. costs for hospitalized cases of simple dengue fever (DF). The ratio of cost per DHF case to cost per hospitalized DF case is 15 . 10 ∕ 13 . 50 = 1 . 119 for Vietnam in Nguyen and Luong [55] cited in [56] Table 1, for lost productivity costs only (in 2017USD); 39 . 09 ∕ 31 . 79 = 1 . 230 for children aged 5–15 in Thailand in Anderson et al. [28], for direct and indirect costs to family excluding transportation; and 134 . 70 ∕ 87 . 90 = 1 . 532 for Thailand in Tozan et al. [57] cited in [56] Table 1, also for lost productivity costs only (in 2017USD). These scale-up factors are relatively close and average to 1.294. (Castro Rodríguez et al. gave a much higher scale-up factor of 2306 . 70 ∕ 497 . 90 = 4 . 633 in Colombia (in 2012USD) [58], but their costs also included direct costs to the healthcare system, which are presumably much higher for DHF due to the additional types of aggressive measures required. For consistency of cost types we use only the other three figures.) We can then deduce the costs for DHF cases vs. simple DF cases from the aggregated data on hospitalized cases in [49] using the previously obtained [global] estimates that 27.4% of hospitalized cases are DF while 72.6% are DHF. Then, substituting *h* = 0 . 726, *k* = 1 . 294 and the average cost per hospitalized dengue case for each country as $c_{H}$ into equation (4) in the Methods section yields separate estimates for hospitalized DF and DHF cases. Finally, the average cost per case for non-ADE or ADE dengue cases is computed using the updated costs (in Table 4) and ().

Costs per case for simple acute Zika were estimated using equation (5) described in the Methods section. For this equation, some global estimates were taken following an earlier study [59, Appendix Table 1]. The proportion (18%) of Zika cases which are symptomatic and median duration of symptoms (4.5 days, a rough mean of the figures 3.5 and 6 days listed for different symptoms) were taken from an early report from Micronesia [60]. As a proxy for the proportion of individuals who would seek medical care for a simple acute case we use the proportion (60%) of individuals in a household survey in Venezuela who said they would seek medical care for a suspected dengue case [61]. The remaining two relevant quantities, for which data were available by country, were the cost of a single outpatient care visit and GNI. Outpatient care cost was taken from WHO-CHOICE [62], given in 2010 international dollars, and converted into 2010USD by multiplying by the Purchasing Power Parity (PPP) factors and currency conversion rates from the World Bank’s World Development Indicators [63] . (For Puerto Rico the cost was estimated by multiplying the US cost by a factor of 0.687 reported by the Puerto Rico Statistical Institute for the cost of outpatient healthcare relative to the US mainland.) GNI per capita also came from the World Bank source.

Zika-related costs per case for GBS and CZS in the Latin American countries come from a UN publication [17] (in 2015USD), with the cost per CZS case adjusted as described in the Methods section to account for the estimated 30% of CZS pregnancies that lead to early pregnancy loss or perinatal death [18,19]. The UN report’s estimated cost per CZS case included productivity loss for the mother and then a caregiver. To account for lost lifetime productivity due to early death, lifespan productivity for each country was estimated by multiplying the 2015 average annual household per capita income by the life expectancy using 3% annual discounting. Income figures came from multiple sources, matching those of the UN report for the Latin American countries [64–67].

Peixoto et al., meanwhile, estimated average costs per case in Brazil for simple acute Zika infection and for Zika infection with GBS, both in 2016USD [68]. In 2024USD, these costs are $28.45 and $15,639.28, respectively, the first of which matches the corresponding UN figure very closely (to within 2.2%, see Table 4), but the second of which is only 1/20 of the UN’s for GBS. The UN report applied cost data from the US (converted using a PPP factor as described above), while Peixoto et al. used data directly from Brazil [68].

In the absence of Zika cost data for the five Asian countries, costs were estimated by extrapolating patterns in dengue cost data and the existing Zika data. This involved treating the first eight rows of Table 4 as a matrix, using rows 1–3 and 5 as a basis for the row space and columns 1, 2, 4, 5 as a basis for the column space, and using a rank-4 CUR decomposition as described in the Methods section.

PAHO/WHO only recorded 20 Zika deaths from among over half a million probable cases [69], a CFR of 0.0034% comparable to the 0.0023% CFR estimated by Puntasecca et al. [19]. Sarmiento-Ospina et al. reported four Zika deaths in Colombia (not included in the count of PAHO/WHO) to patients aged 2, 30, 61, and 72 [70], indicating that deaths can occur at any age, even if primarily among the very young or old. Since nearly all of the cost estimate for fatalities comes from lost years of productivity, we approximate the additional cost for Zika fatalities by that for dengue.

To estimate screening costs we draw on the recent study by Coudeville et al. [71,Table S4], who generated screening administration costs (plus productivity-based costs based on time to obtain the screening) specific to each of the ten countries but assumed a global cost of $10 (in 2015USD) per test purchase for “rapid diagnostic tests with identical specificity (99%) but alternative sensitivities (50-70-90%),” as well as a global 5% wastage rate for the tests based on literature reviews. Combining these costs yields the values given in Table 4, with the exception of Vietnam: Turner et al. made a detailed estimate of screening costs for a large-scale, school-based vaccination campaign of 9-year-olds in Vietnam and estimated $9.25 (in 2016USD, $11.71 in 2024USD) per person using IgG ELISA tests [72]. This is 35% lower than the equivalent estimate in Table 4 using an RDT—a notable discrepancy since ELISA tests should be more expensive than RDTs, but in the absence of further data we use the figure based on Turner et al. [72] for Vietnam, and those based on Coudeville et al. [71] for the other nine countries, for either ELISA or RDT screening costs.

For vaccination costs we again use the approach of Coudeville et al. [71,Table S4], which in this case was based on their work in [73], estimating cost of obtaining and administering the vaccine separately, plus a global cost of $20 (2015USD) per dose plus 5% wastage, to generate a cost per dose. Since vaccination requires 3 doses, the cost is then tripled. We also again substituted country-specific data on vaccine purchase prices where it was available, and assumed that the prices given in [74–76] (37.71, 23, and 23.10 per dose in 2016USD) for Brazil, Indonesia, and the Philippines, respectively, represent purchase prices only.

### Disease burden parameters

Disease burden estimates, discussed below, are summarized in Table 5.

**Table 5:** Parameters representing top-level average burdens (in DALYs) for each case type (d1, d2, z2) with various characteristics or outcomes; also, average age (in years) at death due to dengue. Self-managed cases include asymptomatic; ambulatory cases seek treatment; severe nonfatal involve DHF/DSS. See Results section for further detail.

Parm.	Burden of cases that are...	Value in...									
		Brazil	Colo.	Hond.	Mexico	P.R.	Indo.	Mala.	Phil.	Thai.	Viet.
burd_d1a	...self-managed	0.0162									
burd_d1b	...ambulatory	0.0405									
burd_d*c	...hospitalized	0.0517									
burd_d2d	...severe nonfatal	0.0724									
burd_d2e	...fatal	15.656	27.668	24.870	17.256	17.171	21.174	17.190	23.686	23.858	21.111
Average age at death due to dengue		59.86	50.51	48.60	59.29	64.37	50.96	58.17	46.75	53.77	53.44
burd_za	...acute	0.00102									
burd_z2b	...GBS/chronic	0.64	0.9065	0.436	0.743	1.243	0.302	0.6245	0.296	0.8515	0.5435
burd_z2c	...CZS	81.46	83.74	79.71	82.34	86.63	78.57	81.33	77.11	83.27	80.64
burd_z2	...ADE	0.5220	0.5366	0.5108	0.5276	0.5552	0.5034	0.5211	0.4941	0.5336	0.5167

Numerous studies have been published on the disease burden for dengue in countries around the world. We take our baseline estimates from a recent paper by Zeng et al. [77], who reviewed data from multiple studies to develop global estimates of 0.0307 DALYs (11.2 days lost) per ambulatory case and 0.0351 DALYs (12.8 days) per hospitalized case. These figures include only the patient; many other studies also account for time lost by caretakers (usually family members). One study of eight countries cited by the WHO estimated 14.8 lost days per ambulatory case and 18.9 days per hospitalized case including caretakers’ lost time [78]. A study of dengue hemorrhagic fever in Thailand found an average absence from work of 6 days for caretakers [79], almost exactly the difference between 12.8 and 18.9. Another study suggested adding 50% to account for caretakers’ lost time [80]; 12.8 and 18.9 differ by 47.4% while the two values above for ambulatory cases differ by a factor of only 32%. Since it is plausible that hospitalized cases (being more severe) require a caregiver a greater proportion of the time than an ambulatory case does (this assumes implicitly that the primary reason for managing care at home is medical, not financial), we use the WHO-cited expanded burdens (converted to DALYs) in order to include caregivers’ lost time.

We estimate burdens for other nonfatal case types by scaling these baseline values. Self-managed cases, which often go unreported, are more difficult to document. One study estimated the burden per self-managed case by adjusting the burden for ambulatory cases downward, changing the duration from ten days to four [81]. Although our estimate for days lost per ambulatory cases is not precisely ten, we use this approach and scale down the per-case ambulatory burden to 40% for self-managed cases. Similarly, two studies cited a difference of 40% from hospitalized (DF) cases to severe (DHF) cases, from 9.9 or 10 days to 14 days [80,82]. We apply a similar scale-up to estimate per-case burden for DHF.

Finally, we estimate the per-case burden for dengue fatalities by computing years of life lost (YLLs) as the difference between mean age at death and expected lifespan for each country, and then adding the per-case burden for severe cases, assuming that every dengue fatality becomes severe before death occurs. Average age at dengue death, given in Table 5, was computed from the results of the GBD 2019 study [83].

Burden estimates for Zika come primarily from the review by Puntasecca *et al.* [19]. They computed the [global mean] burden of acute Zika infection by multiplying a mean duration of 0.02 years by a disability weight (DW) of 0.051 to obtain 0.00102 YLDs (Years Lost to Disability); we take this value for *burd_za=burd_z1*. ADE Zika cases are assumed to include this burden as well as burdens for Guillain-Barré Syndrome (GBS) and Congenital Zika Syndrome (CZS). Both of these latter sequelae cause some fatalities, so the burdens vary by country as a function of expected lifespan. Puntasecca *et al.* computed the burden of GBS using a DW of 0.296 and a mean duration of 1 year, generating 0.296 DALYs [19]. In addition, the ZIKV GBS CFR (case fatality ratio) of 0.1 multiplies the difference between the expected lifespan and the age at death (for which [19] listed a global median of 72 years). The total burden for a GBS case is then given by equation (6) in the Methods section. We also observe that the overall CFR for Zika is roughly equal to the CFR of GBS multiplied by *prop*_*z*2*b*, meaning no additional mortality term is necessary.

Meanwhile, the burden of CZS included three components: loss to the child, YLDs of the mother due to depression (0.0349DALYs/case), and YLDs for the child’s caretaker in case of survival (16.83YLDs/case). Loss to the child is the entire lifespan in case of death from CZS (the CFR is 0.299), and lifelong severe disability (with DW 0.795) in case of survival. Substituting these values into equation (7) simplifies this component of the burden to *burd*_*z*2*c* = 16 . 865 + 0 . 856*L*, where *L* is the expected lifespan for the given country.

This makes the overall average burden per ADE Zika case


$$\begin{array}{c} burd\text{\_}z2=burd\text{\_}za+prop\text{\_}z2b\;*\;burd\text{\_}z2b+prop\text{\_}z2c\;*\;burd\text{\_}z2c. \end{array}$$ (14)

We observe that *burd_z2c* (and consequently *burd_z2*) increases as (if) $L_{CZS}$ decreases from *L*, since a reduced lifespan corresponds to more years lost to CZS.

Although screening and vaccination require time for both the prospective vaccinee and a healthcare worker, the economic impact of which (e.g., lost work time) can be incorporated into economic cost models, tables of disability weights do not include actual medical care or preventive care, so we do not incorporate any burden corresponding to screening and vaccination in our model.

Although many sources suggest that individuals with CZS or microcephaly may have reduced expected lifespans, no primary data were found to estimate such a reduction (some modeling studies have assumed 35 years without elaboration or justification, e.g., [84], while others have assumed unaltered lifespans, e.g., [85]). In the absence of data, this study makes the assumption of an unaltered lifespan, noting that the alternative only increases the already high burden of a severe Zika case. The normalized forward sensitivity index of the burden of a severe Zika case (or of a CZS case) ranges from -0.135 to -0.131 among the ten study countries.

## Effects of ADE and vaccination

### Effects of ADE on per-case cost and burden without vaccination

Table 6 presents the average per-case cost and burden by ADE status, computed via (2) as described in the Methods section (as well as the estimated costs for screening and vaccination). In reviewing this table and the ones that follow, it is more productive to focus on trends rather than singling out particular individual values. For instance, one can observe immediately that the economic costs vary more widely than the disease burdens: the range of costs for each type of dengue case spans about 4 times the mean, and the range of costs for each type of Zika case spans 1 to 2 times the mean, while the per-case burdens span 30% or less of the mean value, except for ADE of dengue cases, the range of which does span 1.5 times its mean. This suggests that overall medical and productivity costs vary much more from country to country than does the impact of a case on human lives. Two countries also stand out as obvious opposite extremes: Puerto Rico has by far the highest per-case cost for all case types, the highest costs for screening and vaccination, and the highest per-case disease burden for ADE of Zika; while Vietnam has, also by far, the lowest per-case economic cost for all case types, screening, and vaccination, and the lowest per-case burden for ADE of dengue. Puerto Rico’s status as a U.S. territory, with an economy based on the dollar, may account for a higher overall cost of living (especially as seen through the lens of exchange rates), while Vietnam is known for having low healthcare costs, although these estimates do not address the documented limited availability of medical supplies in some locations.

**Table 6:** Estimated average costs (in 2024 USD) and burdens (in DALYs) per dengue (D) or Zika (Z) case, or per dengue screening or vaccination series. For each virus, 1 signifies without ADE and 2 with ADE.

	Value in...									
**Parm.**	Brazil	Colo.	Hond.	Mexico	P.R.	Indo.	Mala.	Phil.	Thai.	Viet.
cost_D1	453.73	237.21	66.55	522.79	2153.36	136.87	540.12	138.17	200.89	45.79
cost_D2	3406.51	3274.19	1077.18	2504.86	9162.34	668.97	1512.02	1337.33	1179.97	135.07
cost_Z1	20.39	16.56	6.88	28.70	60.60	11.15	27.60	9.74	16.56	9.51
cost_Z2	5658.12	4295.20	1163.22	5025.63	7644.93	1753.92	6309.59	1322.02	3687.54	1287.11
cost_scr	18.14	17.22	15.79	18.14	24.01	16.31	18.40	16.05	17.22	11.71
cost_vac	229.98	146.55	121.19	164.28	267.16	138.16	166.40	134.21	144.08	118.84
burd_D1	0.03541	0.03395	0.03258	0.03390	0.03355	0.03495	0.03345	0.02865	0.03384	0.03905
burd_D2	0.1882	0.3087	0.3120	0.1041	0.08680	0.09252	0.08169	0.1777	0.09521	0.07773
burd_Z1	0.00102									
burd_Z2	0.5220	0.5366	0.5108	0.5276	0.5552	0.5034	0.5211	0.4941	0.5336	0.5167

One can also see that, while ADE of dengue amplifies both per-case cost and burden by a factor of between 2 and 16, ADE of Zika amplifies cost and burden by more than two orders of magnitude: by a factor of about 200 for cost and 500 for burden. This effect comes primarily from the very serious consequences of Zika, associated here to ADE cases, which can kill either the patient (in the case of Guillain-Barré syndrome) or a newborn infant (in the case of congenital Zika syndrome). In fact, the cost and burden of one ADE Zika case far outstrip those of one ADE dengue case, except for economic cost in Puerto Rico. This result highlights the importance of accounting for possible ADE of Zika in estimating the impact of dengue exposure and vaccination.

Next, we can compute population-level weighted average per-infection cost and burden for each virus at the outset of an outbreak as a function of the background level of dengue seropositivity (prior exposure) via (8), as described in the Methods section, distinguishing scenarios where the current and prior dengue serotypes match or differ. The resulting weighted averages are presented in Table 7. It is immediately evident that whether the dengue serotype in circulation is the same as that which produced prior seropositivity makes a huge difference in severity and cost of the average dengue case at the beginning of an outbreak—in Honduras, there is nearly 50 times the cost and 30 times the burden when the serotypes differ—especially since the assumed prior exposure levels in these dengue-endemic countries are high (over 70%). Under these weighted averages, the effect of ADE makes per-infection values for Zika higher than for dengue (except, again, for cost in Puerto Rico) regardless of dengue serotype match. Across countries, the same extremes persist: Puerto Rico has the highest per-infection economic costs of all three types and the highest per-infection Zika burden, while Vietnam has the lowest per-infection economic costs and the lowest per-case dengue burden when serotypes differ. Curiously, Vietnam does also have the highest per-case dengue burden when the current and prior serotypes match. The Philippines has the lowest per-infection dengue burden if serotypes match and Zika burden.

We recall that as the prior dengue exposure level (proportion) varies from 0 to 1, the per-exposure cost and burden vary linearly from the per-case non-ADE value to either the ADE value (for Zika and for dengue with different serotypes) or 0 (for dengue with the same serotype). The values given in Table 7, which represent the relatively high background levels reported in the respective regions, are closer to the latter values than to the per-case non-ADE values reported in Table 6. We also observe that the data in Tables 3–7 are completely independent of choice of vaccine (except vaccination costs in Tables 4 and 6, based on CYD-TDV since no cost estimates for TAK-003 were found), and the data in Tables 3–6 do not assume that all non-ADE cases are primary and all secondary cases are ADE, so that readers interested in exploring alternative assumptions can generate corresponding estimates, as illustrated by the example at the end of the Results section.

It should also be noted that over the course of any outbreak, dengue seropositivity levels naturally rise as more people recover from dengue infections. Thus the figures above, which can be used at the outset of an outbreak, tend, over the course of the epidemic, toward 0 for dengue (as more people develop immunity to the circulating serotype(s)) and toward the per-ADE-case value for Zika. A more nuanced tracking of cases and costs requires a model of infection dynamics, such as those of [86–88].

**Table 7:** Estimated weighted-average per-exposure economic costs (in 2024 USD) and disease burdens (in DALYs) for dengue (where the circulating serotype is either the same as (DS) or different than (DD) that of any prior exposure) and Zika, based on regional prior exposure levels from [21,22], in the absence of dengue vaccination.

Qty.	Value in...									
	Brazil	Colo.	Hond.	Mexico	P.R.	Indo.	Mala.	Phil.	Thai.	Viet.
DS cost	113.89	59.54	16.70	131.22	540.49	38.60	152.31	38.96	56.65	12.91
DD cost	2665.37	2511.91	823.51	2007.36	7403.09	518.92	1237.95	999.17	903.87	109.89
Zika cost	4243.05	3221.26	872.98	3771.40	5741.26	1262.46	4538.07	951.96	2652.32	926.83
DS burden	0.008887	0.008521	0.008177	0.008509	0.008420	0.009855	0.009434	0.008080	0.009543	0.01101
DD burden	0.1499	0.2398	0.2418	0.08645	0.07344	0.07628	0.06808	0.1357	0.07790	0.06683
Zika burden	0.3912	0.4022	0.3828	0.3955	0.4161	0.3617	0.3745	0.3550	0.3834	0.3713

### Effects of ADE and vaccination together

The expressions developed in the Methods section for the screening and vaccination cost per person, and impacts per exposure of dengue—same serotype (11), dengue—different serotypes (12), and Zika (13) can all be seen to be linear in *p* (and indeed in each of the other input variables mentioned), reducing to the no-vaccination values given in Table 7 for *p* = 0, so it is enough here to present the opposite endpoint where *p* = 1, in order to see the range and maximum effect of vaccination as compliance varies. (Estimates for intermediate values of *p* can thus be developed as weighted averages of the two extreme values.) Note that the average screening and vaccination cost per person depend on prior dengue exposure, compliance, and screening characteristics, and the Zika per-infection cost and burden depend on those parameters as well as $a_{0}$, the proportion of fourfold vaccine failure, but both of these quantities are independent of circulating and historical dengue serotypes, so only a single value is reported for each, per country.

The values for the four computations mentioned above are presented in Tables C and D in S1 Appendix using data on CYD-TDV. For reference, Table A in S1 Appendix gives values of the underlying vaccination outcome probabilities $a_{0}$, $a_{\omega }$, and $b_{2}$ (the latter two serotype-specific) defined above, based on the vaccine efficacies given in Table 2. Dengue cost and burden in scenarios where the dengue serotype of prior exposure differs from that currently circulating are given in Table D in S1 Appendix, with historical/circulating serotypes given in the first column. Considering two historical pairs of dengue outbreaks, DENV-1 in 1977 followed by DENV-2 in 1981 in Cuba and DENV-3 in 2002 followed by DENV-2 in 2007-8 in the Rio de Janeiro area of Brazil, we can see that the 1/2 combination seen in Cuba ranks near the middle of the possible pairings in terms of cost and burden, while the 3/2 combination seen in Rio ranks higher, near the 75th percentile. More generally we see that the serotype of prior exposure seems to matter more than the currently circulating one when everyone seeks vaccination; this follows from the high background seropositivity levels and the qualitatively larger variation by prior-exposure serotype in lack of effective vaccine protection for seropositives ($1-b_{2}$ with the values above) compared to the effects of circulating serotype ($a_{\omega }$ above, and note $a_{0}$ is serotype-independent).

The typical specificity of an ELISA screening test is slightly lower than that of an RDT (0.975 vs. 0.987) while its sensitivity is considerably higher (0.876 vs. 0.635). Both of these differences increase the numbers of true positives and false positives using an ELISA test, and consequently increase the proportion of screened individuals approved for vaccination, in this case by about 38%. Consequently the mean cost per person [in the population] of screening and vaccination is about 31% higher using an ELISA screener than using an RDT (the difference is less than 38% because everyone screened has to pay for a screening regardless of the outcome). Likewise, the impact (whether for the better or the worse) of vaccination following an ELISA screening on dengue or Zika cost or burden is nearly twice (about 1.9 times) that of vaccination following an RDT screening.

Dengue vaccination with the characteristics given in Table 2 increases the mean per-infection cost of Zika by less than 1% overall, and the burden by no more than 0.5%, regardless of screener type, because the high dengue seropositivity level (and reasonably good screening) means relatively few dengue-seronegatives are vaccinated. This result is quite consistent across countries: the amplification factor’s range is less than 0.5% of the mean, and typically less than 0.1% of the mean. Therefore, although dengue vaccination does exacerbate Zika case severity, in a highly dengue-endemic area the prior dengue exposure has a much larger impact (with no prior dengue seropositivity, dengue vaccination would instead amplify Zika cost per case by a factor of about 5 using ELISA tests or a factor of about 2.75 using RDTs, and Zika burden by a factor of about 12.5 using ELISA tests or about 6.5 using RDTs).

The impact of screening and vaccination on per-infection cost and burden for dengue is more complex, as indicated in earlier discussion. First, whether the circulating and historical dengue serotypes match or not remains a much larger determinant than vaccination of the magnitude of the cost or burden of an average infection. When they are the same, vaccination changes the mean per-infection cost and burden by less than 2.5%, and typically less than 1%, either up or down. When the serotypes differ, however, vaccination can reduce per-infection cost and burden dramatically, by up to 49–83% depending on the scenario (screener, serotypes, and cost vs. burden). As noted earlier, this difference follows from the fact that seropositivity prevents further dengue infection by the same serotype, making vaccination less relevant, but primes a person for ADE of a second infection by a different serotype, making vaccination a “game-changer.”

There are some smaller trends across serotypes, screeners, and countries. When the circulating and historical dengue serotype are the same, vaccination slightly increases average per-infection dengue cost in Colombia and Honduras for all serotypes, and burden for serotypes 1 and 2. It also slightly increases cost in the Philippines for serotypes 1 and 2. For all other combinations of screener, serotype, and country, vaccination slightly reduces cost and burden. Colombia and Honduras also see the greatest reduction in per-infection dengue cost and burden when the serotypes differ, with the maximum (*p* = 1) reduction exceeding 80% when using an ELISA screener (the per-infection cost can also drop by over 80% in Brazil and the Philippines when using an ELISA test and prior exposure is to serotype 1 or 2). In general, when the serotypes differ, vaccination reduces both cost and burden per infection by up to 73–80% using an ELISA screener, and 53–58% using an RDT. These results are consistent across countries: the range of the maximum change is only 10–16% of the mean. Since an ELISA-based screening tends to lead to more vaccinations under our estimates, the consequent higher cost of the vaccination program is accompanied by a proportionate increase in savings (for both cost and burden per infection) compared to use of an RDT. In Brazil, for instance, while the increased likelihood of screening approving vaccination raises the average cost per screening (and vaccination if approved) by more than 43 2024USD, and thus the average cost per Zika infection by over 16 2024USD (if everyone seeks screening), and reduces the cost per average dengue infection in the dengue—same serotype scenario by less than 0.6 2024USD, it can also reduce the cost per dengue infection by nearly 600 2024USD in the dengue—different serotypes scenario (again if everyone seeks vaccination).

Note that the qualitative results above for the dengue—same serotype scenario are independent of the initial dengue seropositivity level even though it affects the absolute cost and burden, because 1 - *α* factors out of the entire expression (11). Also, as the seropositivity level drops toward 0, the values for the dengue—different serotypes scenario approach those for the dengue—same serotype scenario, since without prior exposure, the historical serotype becomes irrelevant. Finally, on the other hand, dengue seropositivity builds over the course of an outbreak, so the per-infection estimates given here, which might apply toward the beginning of an outbreak, would change as infection (or dengue vaccination) makes an individual immune to reinfection by the same virus but primes that individual for ADE of a subsequent infection by the other virus.

The framework described here can be applied to other scenarios than those for which estimates are given in this section. For instance, in order to estimate cost per case under an assumption that 60% of the population seek screening and vaccination, since per-exposure estimates vary linearly in the proportion *p*, linear interpolation gives 60% times the value under full compliance (*p* = 1, Tables C–D in S1 Appendix) plus 40% of the value without vaccination (*p* = 0, Table 7). (Per-infection estimates for dengue would require first estimating the per-exposure value as above, and then rescaling via (9).) As another example, a reader who wishes to consider a scenario where secondary dengue cases are half ADE, half subclinical could cut per-case cost and burden estimates for ADE dengue cases in half to estimate values for an average secondary case. In a third example, a hypothesis of lower prior dengue exposure, say *α* = 25*%*, and no vaccination would generate per-exposure estimates that are 75% of the non-ADE value plus 25% of the value for dengue-seropositives (which is the ADE value except in the case of dengue when the historical and current serotypes are the same when it’s 0 instead). To incorporate several such alternate hypotheses simultaneously, one can substitute all the desired parameter values into equation (11), (12), or (13) for a specified country and ADE status (to select values from Table 6), screener type, vaccine, and (as applicable) circulating dengue serotype (to select values from Table 2).

Finally, to incorporate also combinations of multiple dengue serotypes past and/or present (as at the end of the Methods section), one must start with per-case values (obtained as described above or from Tables C–D in S1 Appendix) for each possible combination (historical and current) of dengue serotypes, and then use the desired prior and current serotype distributions to generate a weighted average. A scenario where 20% of the population has prior exposure to DENV-1, another 25% has prior exposure to DENV-2, 5% more have prior exposure to both, and the remaining 50% have no prior dengue exposure, and meanwhile the only circulating serotype is DENV-2, would require using the total prior seropositivity rate *α* = 0 . 5 in generating per-exposure values (say $v_{1}$ and $v_{2}$, respectively) for separate scenarios where prior exposure is to DENV-1 or to DENV-2 (to identify $b_{2}$) and DENV-2 is circulating (to identify $a_{\omega }$), and then taking the weighted average $(20\%v_{1}\;+\;25\%v_{2}\;+\;5\%\;\cdot \;0)∕50\%$. If in addition not only DENV-2 but also DENV-3 is circulating, with 75% of new infections being DENV-2 and 25% being DENV-3, then the overall per-exposure cost or burden is 75% of the value derived above (where DENV-2 alone circulates) plus 25% of the analogous value where DENV-3 alone circulates.

It is also possible to substitute the efficacies reported for the TAK-003 vaccine (in Table 2) in lieu of those for CYD-TDV. Tables E and F in S1 Appendix, for example, give the values analogous to those in Tables C and D in S1 Appendix, but for TAK-003 in a scenario without screening. The values for this scenario (both cost and burden) show a significant difference from those for CYD-TDV with screening, but most of the difference can be attributed to the lack of screening (*ψ* = 1 , *χ* = 0) rather than differences in vaccine efficacies. For instance, in Brazil, cost and burden per exposure for dengue are 70–79% lower (for TAK-003 without screening, compared to CYD-TDV with ELISA-based screening) if DENV-2 is not the circulating serotype in the dengue—same serotype scenario or if DENV-2 is the historical serotype in the dengue—different serotypes scenario, and 92–98% lower otherwise (since TAK-003’s efficacy against DENV-2 is so high); on the other hand, cost and burden per Zika case (already 100 times that of a dengue exposure) are 32% higher. However, if we retain screening with TAK-003 (using ELISA values for *ψ* and *χ*), cost and burden per dengue exposure drop by no more than 2% in the dengue—same serotype scenario, cost and burden per Zika case rise by 1/100 of 1%, and the effects in the dengue—different serotypes scenario depend on the historical serotype: figures drop by 12–14% for DENV-1, rise by 3% for DENV-2, and drop by 22-28% for DENV-3 and DENV-4. In general, removing a screening requirement increases the overall net effect of vaccination, which is to reduce overall dengue toll (despite causing ADE in some individuals, cf. negative efficacies reported in Table B in S1 Appendix) but always to increase Zika toll via ADE. This was true of CYD-TDV as well, but the imperative to avoid doing harm led to the screening recommendation.

## Discussion

This study estimated average (i) per-case cost and burden for dengue and Zika as functions of ADE status and (ii) per-infection values using prior dengue seropositivity levels and vaccination campaign characteristics to estimate the initial proportion of cases involving ADE in a dual outbreak, across two types of screening test, ten countries, and all four dengue serotypes. Our estimates indicate that ADE of disease amplifies per-case cost and burden by 2–16 for dengue but by more than two orders of magnitude for Zika, making any potential role played by dengue seropositivity (including by vaccination) on ADE of Zika a critical factor in estimating the toll of a dual outbreak. We observed a severalfold variation of cost across countries, but only a 30% variation in burden, indicating that economic factors vary more than the underlying socioclinical impact. Puerto Rico and Vietnam are absolute extremes (high vs. low) for all costs, and for one per-ADE-case burden each, while dengue vaccination appears to affect per-infection cost and burden slightly more in Colombia and Honduras than in other countries.

The characteristics of ELISA-based testing imply that it will lead to more vaccinations (correctly and incorrectly) than RDTs. Consequently, use of ELISA screeners increases the cost of a vaccination campaign by about 31% compared to one using RDT screeners; however, it also similarly amplifies the effects of a campaign (on per-infection cost and burden).

Without vaccination, the mean per-infection cost and burden for dengue are an order of magnitude higher (a factor of up to 50 for cost and 30 for burden) when the new outbreak involves a different serotype than that involved in the population’s [observed high] prior exposure, due to the nature of the immune interactions between serotypes. By the same token, dengue vaccination then has a much greater potential to reduce per-infection cost and burden in the latter scenario, by as much as 80% in some countries (using ELISA-based testing), compared to less than 2.5% when the outbreak involves the same dengue serotype.

Dengue vaccination exacerbates a Zika outbreak via ADE by hypothesis, but our estimates indicate that the extremely high (over 70%) pre-existing dengue seropositivity levels in the study countries make the additional effect of dengue vaccination minimal, increasing per-infection cost and burden by less than 1%. In a setting with no prior dengue seropositivity, however, dengue vaccination as modeled here could increase (via ADE) per-infection Zika cost by a factor of up to 5 and burden by a factor of up to 12.5. The framework presented in the Methods section allows interested readers to generate their own estimates by choosing alternate values for parameters.

Public health implications of this study include a need to verify the extent to which ADE plays a role in the more severe complications of Zika infection, since those complications drive some of the trends seen in the per-case cost and burden estimates. In addition, when screening is tied to dengue vaccination, the nature of the screener can have a significant impact on the cost and also the impact of the vaccination campaign. Patterns in which dengue serotypes circulate in endemic areas should also play an important role in vaccination recommendations: when a significant portion of the population has prior exposure to a different serotype than that circulating, average per-case costs can be up to an order of magnitude higher (completely aside from the number of cases), but it is in this setting that an ELISA-based screener, for instance, which approves vaccination for more people can also cut the mean per-case cost by more than half.

The scarcity of data measuring key quantities in determining cost and burden imply significant uncertainty in many if not most of the estimates produced in this study. In particular, the proportions of Zika cases involving complications came from a single study, and the costs of those complications in the Asian countries had to be extrapolated from trends in dengue costs and (based on a single source) for those costs in Latin America. The dominance of cost and burden per ADE Zika case over other case types thus implies potentially great uncertainty in the total cost of an outbreak. The qualitative trends identified here across countries, serotypes, and screeners are less affected by such uncertainties than the absolute numbers. Better data are clearly needed to measure the proportions, costs, and burdens of dengue and (especially) Zika complications.

The most important limitation of this study is that the estimates only describe one average case, primarily at the outset of an outbreak, and not the size of an outbreak. Proper population-level estimates of the total size (including cost and burden) of an outbreak require a model of transmission dynamics, as has been observed by other articles calling for such models (e.g., [8]). Such work is already underway. Since the primary aim of vaccines is to reduce the numbers of cases, such estimates are a necessary prerequisite to comparing the impacts of different vaccines (e.g., CYD-TDV vs. TAK-003).

It is also worth noting that the calculations in this study make use of dengue seropositivity and its hypothesized role in ADE, but do not take into account any prior Zika seropositivity. This may not be a significant limitation, however: one study in French Polynesia and Fiji found that Zika seropositivity declined significantly in adults (but not children) over a two-year period following an outbreak, while dengue seropositivity did not [89]. This result suggests that long-term seropositivity for dengue only, and not for Zika, is likely to play an important role in transmission in areas where Zika has not circulated recently. Models of transmission dynamics can track the potential effects of Zika seropositivity acquired during an outbreak like those assumed here for prior dengue exposure.

Without any cost information for the TAK-003 vaccine, we have primarily used data for CYD-TDV to illustrate the framework. As the TAK-003 vaccine is rolled out, it is anticipated that such data will become available, and the corresponding estimates more important. Since serotype-specific efficacies for TAK-003 are in some cases higher and in other cases lower than those for CYD-TDV, the estimated impact of TAK-003 vaccination compared to that of CYD-TDV vaccination is serotype-dependent; however, if TAK-003 is administered without screening for dengue seropositivity, its net effect will be amplified (as would be that for CYD-TDV), reducing per-exposure dengue cost/burden by nearly two orders of magnitude but increasing the much higher cost-burden per Zika case by one third.

Estimates in the tables are limited to a single circulating dengue serotype, and any prior exposure also to a single serotype. However, an example illustrated how these single-serotype estimates can be used to develop multiple-serotype estimates valid near the beginning of an outbreak.

Another potential limitation on accuracy is our not distinguishing here between serotype-specific vaccine efficacies for dengue-seropositives versus seronegatives, but the Phase III trials for the CYD-TDV and TAK-003 vaccines reported some serostatus-specific efficacies with negative values (see Table B in S1 Appendix, cf. [90]), which do not lead to meaningful estimates. We therefore used the data pooled across serostatus for ages 9+. Finally, more study is needed to verify associating the severest consequences of each virus with ADE, especially for Zika, which has been much less studied than dengue.

## Supporting information

S1 Appendix Additional tables. Supplemental parameter values. Cost and burden estimates.
