# Supplementary material for: Estimating per-infection cost and burden for dengue and Zika as a function of antibody-dependent enhancement
Source: PLoS Negl Trop Dis. 2025 Feb 27;19(2):e0012876. doi: 10.1371/journal.pntd.0012876 (PMC11906165; doi:10.1371/journal.pntd.0012876)
Supplement: S1 Appendix — Supplemental parameter values. Cost and burden estimates. [file pntd.0012876.s001.pdf]

Supplementary Information for "Estimating per-infection cost and burden for dengue and Zika as a function of antibody-dependent enhancement" by Christopher M. Kribs

S1 Appendix: Additional tables

Table A: Compound parameters derived from serotype-specific vaccine efficacies

		a_ω for circulating serotype			
Setting [source]	a_0	1	2	3	4
CYD-TDV, Latin America [1]	0.01663	0.1174	0.1221	0.08695	0.07634
CYD-TDV, southeast Asia [2]	0.01734	0.1251	0.1331	0.07954	0.08961
TAK-003 [3]	0.0008325	0.03819	0.005156	0.03913	0.03909

	b_2 for historical serotype			
Setting [source]	1	2	3	4
CYD-TDV, Latin America [1]	0.9665	0.9712	0.9361	0.9254
CYD-TDV, southeast Asia [2]	0.9653	0.9733	0.9197	0.9298
TAK-003 [3]	0.9968	0.9638	0.9978	0.9977

Table B: Transmission-related quantities with multiple values in the literature, not directly reported in Table 2 in the main text. * first 25 months, ages 9–16.

Quantity	Value	Ref.
Dengue transmission probability,	0.6 (Brazil, 2010)	[4]
mosquito to human	0.675 (DENV-1, Thailand, 2011)	[5]
	0.745 (DENV-2, Thailand, 2011)	[5]
Dengue transmission probability,	1 (Brazil, 2010)	[4]
human to mosquito	0.934 (DENV-1, Thailand, 2011)	[5]
	0.903 (DENV-2, Thailand, 2011)	[5]
ZIKV vectorial capacity of *Aedes aegypti*	0.2 (viral conc. 10^5/mL)	[6]
	0.35 (viral conc. 10^6/mL)	[6]
Zika viral load amplification factors	60–248	[7]
in vitro due to DENV antibodies	140, 275, up to 200	[8]
Mosquito/human density ratio	0.42	[9]
	0.62	[10]
Aedes aegypti adult lifespan	2–6 weeks	[11]
	27.5 days post feeding	[6]
	27.56 days (Thailand, lab)	[5]
vaccine efficacy, by dengue serotype (1–4)		
CYD-TDV, *, Latin America	0.503, 0.423, 0.740, 0.777	[1, Table 3]
CYD-TDV, *, SE Asia	0.500, 0.350, 0.784, 0.753	[2, Table 3]
CYD-TDV, *, seropositive	0.674, 0.673, 0.800, 0.893	[12, Table S43]
CYD-TDV, *, seronegative	0.409, -0.208, 0.515, 0.651	[12, Table S43]
TAK-003, first year	0.737, 0.977, 0.626, 0.632	[3, Table 2]
TAK-003, first 4.5 years, seropositives	0.561, 0.804, 0.523, 0.706	[13, Figure 2]
TAK-003, first 4.5 years, seronegatives	0.454, 0.881, -0.155, -1.056	[13, Figure 2]

Table C: Estimated weighted-average economic costs (in 2024USD) and disease burdens (in DALYs) for screening and vaccination per person (cost only) and dengue (same serotype scenario) and Zika per exposure, under an assumption of full compliance ($p = 1$) with screening and CYD-TDV vaccination, with regional prior exposure levels from [2, 1], as functions of screening test and (for dengue cases) dengue serotype.

		**Value in...**									
**Qty.**	**Test**	Brazil	Colo.	Hond.	Mexico	P.R.	Indo.	Mala.	Phil.	Thai.	Viet.
Screening and vaccination											
cost	ELISA	170.47	114.29	96.06	126.96	200.97	104.18	124.23	101.41	108.86	87.30
cost	RDT	128.27	87.40	73.82	96.81	151.94	79.80	94.87	77.74	83.44	66.33
Dengue, same serotype											
DENV-1 cost	ELISA	113.60	60.49	17.09	129.84	533.96	38.24	149.91	39.19	56.30	12.72
DENV-1 cost	RDT	113.74	60.03	16.90	130.50	537.10	38.41	151.06	39.08	56.47	12.81
DENV-2 cost	ELISA	113.70	60.58	17.12	129.91	534.23	38.28	149.99	39.26	56.37	12.72
DENV-2 cost	RDT	113.79	60.08	16.92	130.54	537.23	38.43	151.11	39.12	56.50	12.81
DENV-3 cost	ELISA	112.95	59.86	16.88	129.36	532.21	38.02	149.42	38.76	55.92	12.67
DENV-3 cost	RDT	113.40	59.71	16.80	130.25	536.18	38.30	150.81	38.86	56.27	12.79
DENV-4 cost	ELISA	112.72	59.64	16.81	129.19	531.60	38.07	149.53	38.85	56.00	12.68
DENV-4 cost	RDT	113.28	59.59	16.76	130.17	535.87	38.32	150.86	38.91	56.31	12.79
DENV-1 burden	ELISA	0.008807	0.008539	0.008206	0.008377	0.008277	0.009695	0.009274	0.008039	0.009393	0.01081
DENV-1 burden	RDT	0.008846	0.008530	0.008192	0.008440	0.008345	0.009772	0.009350	0.008059	0.009465	0.01091
DENV-2 burden	ELISA	0.008813	0.008548	0.008215	0.008380	0.008279	0.009700	0.009278	0.008049	0.009398	0.01081
DENV-2 burden	RDT	0.008848	0.008535	0.008197	0.008442	0.008347	0.009774	0.009353	0.008064	0.009468	0.01091
DENV-3 burden	ELISA	0.008771	0.008480	0.008146	0.008357	0.008260	0.009665	0.009248	0.007981	0.009362	0.01078
DENV-3 burden	RDT	0.008827	0.008500	0.008161	0.008430	0.008337	0.009756	0.009337	0.008029	0.009449	0.01089
DENV-4 burden	ELISA	0.008759	0.008459	0.008125	0.008350	0.008254	0.009671	0.009253	0.007994	0.009369	0.01079
DENV-4 burden	RDT	0.008820	0.008489	0.008150	0.008427	0.008334	0.009760	0.009340	0.008036	0.009453	0.01090
Zika											
cost	ELISA	4277.84	3247.66	880.11	3802.23	5788.06	1274.53	4581.59	961.05	2677.75	935.68
cost	RDT	4261.14	3234.99	876.69	3787.43	5765.60	1268.74	4560.70	956.68	2665.55	931.43
burden	ELISA	0.3944	0.4055	0.3860	0.3987	0.4195	0.3652	0.3781	0.3584	0.3871	0.3748
burden	RDT	0.3929	0.4039	0.3844	0.3971	0.4178	0.3635	0.3763	0.3568	0.3853	0.3731

Table D: Estimated weighted-average economic costs (in 2024USD) and disease burdens (in DALYs) for dengue (different serotypes scenario) per exposure, under an assumption of full compliance ($p = 1$) with screening and CYD-TDV vaccination, with regional prior exposure levels from [2, 1], as functions of screening test and historical and circulating dengue serotypes.

**Hist./ Circ.**	**Test**	**Value in...** Brazil	Colo.	Hond.	Mexico	P.R.	Indo.	Mala.	Phil.	Thai.	Viet.
Cost											
1/2	ELISA	504.85	436.55	140.81	417.54	1586.30	112.43	317.59	187.50	187.16	27.69
1/2	RDT	1099.28	1007.30	328.54	855.19	3187.87	224.33	571.27	410.74	384.39	50.35
1/3	ELISA	504.10	435.83	140.57	416.98	1584.28	112.18	317.02	186.99	186.71	27.64
1/3	RDT	1098.89	1006.92	328.42	854.90	3186.82	224.19	570.97	410.48	384.16	50.32
1/4	ELISA	503.88	435.61	140.50	416.82	1583.67	112.22	317.13	187.09	186.80	27.65
1/4	RDT	1098.77	1006.81	328.38	854.81	3186.50	224.22	571.03	410.53	384.20	50.33
2/1	ELISA	494.39	426.49	137.50	409.84	1558.15	109.02	309.89	180.69	181.15	27.01
2/1	RDT	1091.71	1000.02	326.15	849.62	3167.52	221.87	565.71	405.82	380.05	49.85
2/3	ELISA	493.74	425.86	137.29	409.36	1556.40	108.81	309.41	180.26	180.77	26.96
2/3	RDT	1091.37	999.70	326.04	849.37	3166.60	221.75	565.45	405.60	379.86	49.83
2/4	ELISA	493.51	425.64	137.22	409.19	1555.79	108.86	309.52	180.36	180.86	26.97
2/4	RDT	1091.26	999.58	326.01	849.29	3166.29	221.78	565.51	405.65	379.90	49.83
3/1	ELISA	572.91	501.96	162.33	467.58	1769.36	131.58	360.87	225.77	220.93	31.56
3/1	RDT	1148.64	1054.73	344.15	891.48	3320.62	238.21	602.66	438.50	408.89	53.15
3/2	ELISA	573.01	502.06	162.36	467.66	1769.63	131.61	360.95	225.85	221.00	31.57
3/2	RDT	1148.69	1054.78	344.17	891.52	3320.76	238.23	602.70	438.54	408.92	53.16
3/4	ELISA	572.04	501.12	162.05	466.94	1767.00	131.41	360.49	225.44	220.64	31.53
3/4	RDT	1148.18	1054.30	344.01	891.14	3319.39	238.13	602.46	438.33	408.73	53.13
4/1	ELISA	596.63	524.76	169.83	485.02	1833.15	127.34	351.29	217.30	213.45	30.70
4/1	RDT	1165.83	1071.26	349.59	904.12	3366.86	235.14	595.71	432.36	403.47	52.53
4/2	ELISA	596.73	524.85	169.86	485.09	1833.41	127.37	351.37	217.38	213.52	30.71
4/2	RDT	1165.88	1071.31	349.60	904.16	3366.99	235.16	595.76	432.40	403.50	52.53
4/3	ELISA	595.98	524.13	169.62	484.54	1831.39	127.12	350.80	216.87	213.07	30.66
4/3	RDT	1165.49	1070.93	349.48	903.87	3365.94	235.03	595.46	432.13	403.27	52.51
Burden											
1/2	ELISA	0.03043	0.04400	0.04404	0.02033	0.01825	0.01995	0.01833	0.02774	0.01995	0.01943
1/2	RDT	0.06332	0.09785	0.09845	0.03855	0.03346	0.03548	0.03205	0.05744	0.03592	0.03250
1/3	ELISA	0.03039	0.04393	0.04397	0.02031	0.01823	0.01992	0.01830	0.02768	0.01992	0.01940
1/3	RDT	0.06330	0.09782	0.09841	0.03854	0.03345	0.03547	0.03204	0.05740	0.03591	0.03249
1/4	ELISA	0.03038	0.04391	0.04395	0.02030	0.01822	0.01993	0.01831	0.02769	0.01992	0.01941
1/4	RDT	0.06329	0.09781	0.09840	0.03853	0.03345	0.03547	0.03204	0.05741	0.03591	0.03250
2/1	ELISA	0.02985	0.04305	0.04308	0.02001	0.01798	0.01948	0.01792	0.02684	0.01947	0.01903
2/1	RDT	0.06290	0.09717	0.09775	0.03832	0.03327	0.03514	0.03175	0.05678	0.03557	0.03222
2/3	ELISA	0.02982	0.04299	0.04302	0.01999	0.01796	0.01945	0.01789	0.02678	0.01944	0.01901
2/3	RDT	0.06288	0.09713	0.09772	0.03831	0.03326	0.03513	0.03174	0.05676	0.03556	0.03221
2/4	ELISA	0.02981	0.04297	0.04300	0.01998	0.01796	0.01946	0.01790	0.02679	0.01944	0.01902
2/4	RDT	0.06288	0.09712	0.09771	0.03830	0.03325	0.03513	0.03174	0.05676	0.03556	0.03221
3/1	ELISA	0.03420	0.05017	0.05027	0.02241	0.01998	0.02260	0.02067	0.03283	0.02268	0.02166
3/1	RDT	0.06605	0.1023	0.1030	0.04006	0.03472	0.03740	0.03375	0.06113	0.03790	0.03412
3/2	ELISA	0.03420	0.05018	0.05028	0.02241	0.01998	0.02261	0.02068	0.03284	0.02268	0.02166
3/2	RDT	0.06605	0.1023	0.10297	0.04006	0.03472	0.03741	0.03375	0.06113	0.03790	0.03413
3/4	ELISA	0.03415	0.05009	0.05019	0.02238	0.01996	0.02258	0.02065	0.03278	0.02265	0.02164
3/4	RDT	0.06602	0.1023	0.1029	0.04004	0.03471	0.03739	0.03374	0.06110	0.03789	0.03411
4/1	ELISA	0.03551	0.05232	0.05244	0.02313	0.02059	0.02202	0.02015	0.03170	0.02207	0.02116
4/1	RDT	0.06700	0.1039	0.1045	0.04058	0.03515	0.03698	0.03337	0.06031	0.03746	0.03377
4/2	ELISA	0.03551	0.05233	0.05245	0.02314	0.02059	0.02202	0.02016	0.03171	0.02208	0.02117
4/2	RDT	0.06700	0.1039	0.1046	0.04058	0.03516	0.03698	0.03337	0.06032	0.03747	0.03377
4/3	ELISA	0.03547	0.05226	0.05238	0.02311	0.02057	0.02199	0.02013	0.03165	0.02204	0.02114
4/3	RDT	0.06698	0.1039	0.1045	0.04057	0.03515	0.03696	0.03336	0.06028	0.03745	0.03375

Table E: Estimated weighted-average economic costs (in 2024USD) and disease burdens (in DALYs) for dengue (same serotype scenario) and Zika per exposure, under an assumption of full compliance ($p = 1$) with TAK-003 vaccination without screening, with regional prior exposure levels from [2, 1], as functions of dengue serotype.

**Measure,**	**Value in...**									
**[Serotype]**	Brazil	Colo.	Hond.	Mexico	P.R.	Indo.	Mala.	Phil.	Thai.	Viet.
Dengue, same serotype										
cost, 1	32.75	31.43	10.34	24.12	88.27	7.24	16.41	14.43	12.75	1.47
cost, 2	4.50	4.29	1.41	3.35	12.31	1.00	2.33	1.98	1.76	0.21
cost, 3	33.55	32.21	10.59	24.71	90.43	7.41	16.81	14.79	13.07	1.50
cost, 4	33.52	32.18	10.58	24.69	90.35	7.41	16.79	14.77	13.05	1.50
burden, 1	0.001812	0.002966	0.002997	0.001005	0.0008390	0.001005	0.0008875	0.001920	0.001033	0.0008463
burden, 2	0.0002511	0.0004067	0.0004106	0.0001418	0.0001194	0.0001427	0.0001266	0.0002651	0.0001464	0.0001222
burden, 3	0.001857	0.003039	0.003071	0.001029	0.0008595	0.001029	0.0009092	0.001967	0.001058	0.0008669
burden, 4	0.001855	0.003036	0.003068	0.001028	0.0008587	0.001028	0.0009083	0.001965	0.001057	0.0008661
Zika										
cost	5656.94	4294.30	1162.97	5024.58	7643.35	1753.51	6308.11	1321.71	3686.67	1286.81
burden	0.5219	0.5365	0.5106	0.5275	0.5550	0.5033	0.5210	0.4940	0.5335	0.5165

Table F: Estimated weighted-average economic costs (in 2024USD) and disease burdens (in DALYs) for dengue (different serotype scenario) per exposure, under an assumption of full compliance ($p = 1$) with TAK-003 vaccination without screening, with regional prior exposure levels from [2, 1], as functions of historical and circulating dengue serotypes.

**Hist./**	**Value in...**									
**Circ.**	Brazil	Colo.	Hond.	Mexico	P.R.	Indo.	Mala.	Phil.	Thai.	Viet.
Cost										
1/2	12.58	12.05	3.96	9.29	34.03	2.53	5.76	5.02	4.44	0.51
1/3	41.63	39.97	13.15	30.65	112.16	8.93	20.25	17.83	15.75	1.81
1/4	41.60	39.94	13.14	30.63	112.07	8.93	20.23	17.81	15.74	1.81
2/1	125.10	120.20	39.54	92.03	336.68	24.62	55.71	49.19	43.42	4.98
2/3	125.91	120.97	39.80	92.62	338.84	24.80	56.11	49.55	43.73	5.01
2/4	125.88	120.94	39.79	92.60	338.76	24.79	56.09	49.53	43.72	5.01
3/1	38.43	36.89	12.13	28.30	103.55	8.31	18.83	16.57	14.64	1.68
3/2	10.18	9.75	3.20	7.53	27.58	2.07	4.74	4.11	3.65	0.42
3/4	39.20	37.63	12.38	28.86	105.63	8.48	19.21	16.91	14.94	1.72
4/1	38.52	36.98	12.16	28.36	103.80	8.32	18.87	16.61	14.67	1.68
4/2	10.28	9.84	3.23	7.60	27.83	2.09	4.78	4.15	3.68	0.43
4/3	39.32	37.75	12.42	28.95	105.96	8.50	19.27	16.96	14.98	1.72
Burden										
1/2	0.0006975	0.0011387	0.0011502	0.0003885	0.0003252	0.0003530	0.0003123	0.0006689	0.0003628	0.0002989
1/3	0.002303	0.003771	0.003810	0.001276	0.001065	0.001239	0.001095	0.002371	0.001275	0.001044
1/4	0.002301	0.003768	0.003807	0.001275	0.001065	0.001238	0.001094	0.002369	0.001274	0.001043
2/1	0.006917	0.011337	0.011455	0.003826	0.003192	0.003409	0.003011	0.006538	0.003508	0.002867
2/3	0.006961	0.011410	0.011528	0.003850	0.003213	0.003434	0.003032	0.006585	0.003533	0.002887
2/4	0.006960	0.011407	0.011526	0.003849	0.003212	0.003433	0.003031	0.006583	0.003532	0.002886
3/1	0.002126	0.003481	0.003517	0.001178	0.000984	0.001152	0.001018	0.002204	0.001185	0.000971
3/2	0.0005650	0.0009214	0.0009307	0.0003153	0.0002641	0.0002906	0.0002572	0.0005491	0.0002986	0.0002464
3/4	0.002169	0.003551	0.003588	0.001202	0.001003	0.001176	0.001039	0.002249	0.001210	0.000990
4/1	0.002131	0.003490	0.003526	0.001181	0.000986	0.001155	0.001020	0.002209	0.001188	0.000973
4/2	0.0005701	0.0009298	0.0009392	0.0003181	0.0002664	0.0002930	0.0002593	0.0005537	0.0003010	0.0002485
4/3	0.002176	0.003562	0.003599	0.001205	0.001007	0.001179	0.001042	0.002256	0.001213	0.000993

References

[1] Villar L, Dayan G, Arredondo-García J, Rivera D, Cunha R, Deseda C, et al. Efficacy of a tetravalent dengue vaccine in children in Latin America. New England Journal of Medicine 2015; 372(2): 113–123. doi:10.1056/NEJMoa1411037

[2] Capeding M, Tran N, Hadinegoro S, Ismail H, Chotpitayasunondh T, Chua M, et al. Clinical efficacy and safety of a novel tetravalent dengue vaccine in healthy children in Asia: a phase 3, randomised, observer-masked, placebo-controlled trial. Lancet 2014; 384(9951): 1358–1365. doi:10.1016/S0140-6736(14)61060-6

[3] Biswal S, Reynales H, Saez-Llorens X, Lopez P, Borja-Tabora C, Kosalaraksa P, et al. Efficacy of a tetravalent dengue vaccine in healthy children and adolescents. New England J Med 2019; 381: 2009–2019. doi: 10.1056/NEJMoa1903869

[4] Massad E, Coutinho FAB, Burattini MN, Amaku M. Estimation of R_0 from the initial phase of an outbreak of a vector-borne infection. Tropical Medicine and International Health 2010; 15(1): 120–126. doi:10.1111/j.1365-3156.2009.02413.x

[5] Lambrechts L, Paaijmans KP, Fansiri T, Carrington LB, Kramer LD, Thomas MB, et al. Impact of daily temperature fluctuations on dengue virus transmission by *Aedes aegypti*, Proc Natl Acad Sci U S A 2011 May 3; 108(18): 7460–7465. doi: 10.1073/pnas.1101377108

[6] Tesla B, Demakovsky LR, Packiam HS, Mordecai EA, Rodríguez AD, Bonds MH, et al. Estimating the effects of variation in viremia on mosquito susceptibility, infectiousness, and R_0 of Zika in *Aedes aegypti*. PLoS Neglected Tropical Diseases 2018; 12(8): e0006733. doi:10.1371/journal.pntd.0006733

[7] Charles A, Christofferson R. Utility of a dengue-derived monoclonal antibody to enhance Zika infection in vitro. PLoS Currents 2016; 8: currents.outbreaks.4ab8bc87c945eb41cd8a49e127082620.

[8] Paul LM, Carlin ER, Jenkins MM, Tan AL, Barcellona CM, Nicholson CO, et al. Dengue virus antibodies enhance Zika virus infection. Clinical and Translational Immunology 2016 Dec 16; 5(12): e117. doi: 10.1038/cti.2016.72.

[9] Rodrigues MM, Monteiro Marques GRA, Nunes Serpa LL, de Brito Arduino M, Voltolini JC, Laurindo Barbosa G, et al. Density of *Aedes aegypti* and *Aedes albopictus* and its association with number of residents and meteorological variables in the home environment of dengue endemic area, São Paulo, Brazil. Parasites and Vectors 2015; 8: 115. doi:10.1186/s13071-015-0703-y

[10] Maciel-de-Freitas R, Eiras AE, Louren co-de-Oliveira R. Calculating the survival rate and estimated population density of gravid *Aedes aegypti* (Diptera, Culicidae) in Rio de Janeiro, Brazil. Cadernos de Saúde Publica 2008; 24(12): 2747–2754.

[11] Tesla B, Demakovsky LR, Mordecai EA, Ryan SJ, Bonds MH, Ngonghala CN, et al. Temperature drives Zika virus transmission: evidence from empirical and mathematical models. Proc. Royal Soc. B 2018; 285: 20180795. doi:10.1098/rspb.2018.0795

[12] Sridhar S, Luedtke A, Langevin E, Zhu M, Bonaparte M, Machabert T, et al. Effect of dengue serostatus on dengue vaccine safety and efficacy, New England Journal of Medicine 2018; 379(4): 327–340. doi: 10.1056/NEJMoa1800820

[13] Tricou V, Yu D, Reynales H, Biswal S, Saez-Llorens X, Sirivichayakul C, et al. Long-term efficacy and safety of a tetravalent dengue vaccine (TAK-003): 4.5-year results from a phase 3, randomised, double-blind, placebo-controlled trial, Lancet Global Health 2024; 12: e257–270.
